# A Staged Whole-Blood Transcriptomic Framework Identifies a Compact Myeloid–Lymphoid Activity Score in Systemic Lupus Erythematosus

**DOI:** 10.3390/genes17060663

**Published:** 2026-06-06

**Authors:** Chuanwei Zhang, Lijun Pang, Ziheng Zhu, Jianing Wang, Chuanbing Huang

**Affiliations:** 1 Department of Rheumatology, The First Affiliated Hospital of Anhui University of Chinese Medicine, Hefei 230038, China; 2 Center for Xin’an Medicine and Modernization of Traditional Chinese Medicine of IHM, Hefei 230012, China

**Keywords:** systemic lupus erythematosus, whole-blood transcriptomics, molecular endotypes, compact transcriptomic score, immune-state stratification, BloodGen3

## Abstract

Background/Objectives: Peripheral-blood transcriptomic profiling can capture molecular heterogeneity in systemic lupus erythematosus (SLE), but discovery-stage signatures often show limited transportability across cohorts and validation layers. This study aimed to establish a staged whole-blood transcriptomic framework and to derive a compact, biologically interpretable activity score. Methods: Public whole-blood bulk transcriptome cohorts were organised into discovery, public validation, and single-cell reference layers. Local orthogonal validation included a peripheral blood mononuclear cell (PBMC) reverse transcription quantitative PCR (RT-qPCR)/flow-cytometric cohort and an expanded whole-blood RT-qPCR validation set. Discovery-stage BloodGen3 profiling included 233 samples, comprising 170 SLE and 63 healthy controls, and endotype discovery was restricted to SLE samples. Candidate genes were compressed into two 6-gene panels, with final selection adjudicated through staged public validation. Results: Two working whole-blood endotypes were identified, characterised by lymphoid versus myeloid/neutrophil-inflammatory polarisation. Although pre6-any showed a marginal discovery-stage advantage, the predefined integrated public-stage adjudication favoured pre6-balanced (*MMP9*, *MYL9*, *HAL*, *CTLA4*, *CD40LG*, *VPREB3*), which was locked as the final panel. In the PBMC cohort, the locked score discriminated SLE from healthy controls (AUC 0.838) and high from low/moderate disease activity (AUC 0.719), with associations with SLEDAI, complement C3/C4, and monocyte subpopulations. In the expanded whole-blood validation set, the score showed SLE-versus-HC discrimination (AUC 0.888, 95% CI 0.821–0.954), high versus low/moderate activity discrimination (AUC 0.918, 95% CI 0.831–0.980), and association with SLEDAI (ρ = 0.819, *p* = 1.25 × 10^−15^). Conclusions: This staged framework yielded a compact myeloid–lymphoid activity score supported across public and local validation layers. The score should be interpreted as a research-grade relative activity score and warrants prospective evaluation in SLE.

## 1. Introduction

Systemic lupus erythematosus (SLE) is a clinically and biologically heterogeneous autoimmune disease characterised by variable organ involvement, immune dysregulation, and disease course [1,2]. This heterogeneity limits the ability of conventional clinical or serological measures to fully resolve underlying molecular variation [3,4]. Peripheral-blood transcriptomic profiling has emerged as an attractive strategy for capturing disease-relevant differences, with increasing translational relevance for molecular classification, biomarker development, and disease-activity contextualisation [3,4,5,6].

Despite this promise, molecular subgroup structures identified in discovery datasets often lack stability across platforms, independent cohorts, and downstream validation layers [3,4,5,7]. This problem is particularly evident when feature selection, biological interpretation, and validation are not clearly separated. Single-cell data can improve cellular interpretability but should not automatically displace robust whole-blood discovery when the primary aim is cross-cohort stratification [8,9]. A translationally useful blood-based score, therefore, requires not only biological plausibility but also a study design that maintains a clear boundary between discovery, adjudication, and validation [4,6].

To address this challenge, we designed a strictly staged framework for peripheral-blood transcriptomic stratification in SLE. Discovery was restricted to public whole-blood bulk transcriptome cohorts. Candidate compression and final panel selection were adjudicated through independent public whole-blood validation cohorts. Single-cell analysis was used solely for biological anchoring, and local orthogonal validation comprised a PBMC RT-qPCR/flow-cytometric cohort and an expanded, additional whole-blood RT-qPCR validation set. Our aims were: to identify working whole-blood transcriptomic endotypes in SLE; to derive and formally lock a compact transcriptomic score; and to assess whether the locked score retained biological interpretability and local clinical relevance across distinct validation layers. The novelty of the present work lies not in the identification of previously unknown immune programmes—type I interferon-inducible and myeloid/neutrophil-associated signatures have been extensively characterised in SLE—but rather in the staged analytical design itself: final panel selection was adjudicated through public-stage validation before any local testing, single-cell data were reserved for biological anchoring only, and local validation cohorts were used for orthogonal confirmation without reverse refinement of the locked panel. This design was intended to reduce the risk of circular feature selection that commonly limits the transportability of transcriptomic biomarker studies.

## 2. Materials and Methods

### 2.1. Study Design and Cohort Architecture

**Figure 1:**
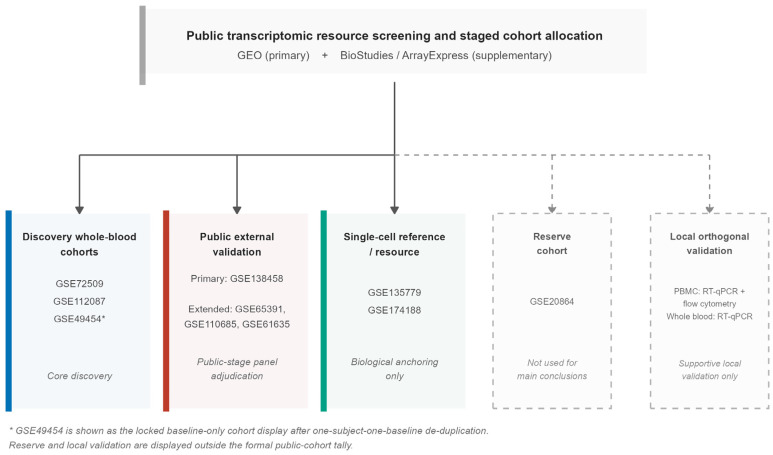
Study architecture and staged cohort allocation. Public transcriptomic resources were allocated into discovery whole-blood cohorts, public external validation cohorts, a single-cell reference/resource layer, a reserve cohort, and local orthogonal validation layers. The local validation component included a PBMC-based RT-qPCR and flow-cytometric validation cohort and an expanded, additional whole-blood RT-qPCR validation set. GSE49454 is displayed at the architecture level as 78 baseline-level samples; downstream discovery analyses used the stricter locked analytic subset (56 samples; total discovery analysis set: 233 samples). The reserve cohort and local validation layers are displayed outside the formal public-cohort tally. Abbreviations: PBMC, peripheral blood mononuclear cell; SLE, systemic lupus erythematosus.

This study used a staged multi-layer design integrating public transcriptomic resources with local orthogonal validation. Public datasets were retrieved from the Gene Expression Omnibus (GEO; with supplementary screening in BioStudies and ArrayExpress) and organised into: discovery whole-blood cohorts, public external validation whole-blood cohorts, a single-cell reference/resource layer, and a reserve layer. Local orthogonal validation comprised a PBMC-based RT-qPCR and flow-cytometric validation cohort and an expanded, additional whole-blood RT-qPCR validation set. The public resource set comprised three discovery cohorts, one primary validation cohort, three extended validation cohorts, and two single-cell reference/resource datasets; a reserve cohort was listed separately (Figure 1 and Table S1).

### 2.2. Public Cohort Preprocessing and BloodGen3 Aggregate-Level Profiling

Discovery was restricted to three public whole-blood bulk transcriptome cohorts (GSE72509, GSE112087, GSE49454), each processed separately with cohort-specific normalisation, log-transformation where appropriate, gene-symbol harmonisation, and duplicate-gene collapsing. No unified cross-cohort batch correction was applied prior to compact-score computation; instead, each gene was z-standardised to zero mean and unit variance within each cohort independently (cohort-wise z-standardisation), ensuring that the compact score reflects relative within-cohort expression variation rather than cross-cohort absolute differences (see Supplementary Methods S1 for full preprocessing details). The final discovery analysis set comprised 233 samples (170 SLE, 63 HC); GSE49454 contributed 56 samples after locked analytic-subset filtering (architecture-level display: 78 samples). Blood transcriptional activity was quantified using the BloodGen3 aggregate-level framework [10,11,12]. Single-sample aggregate scores were generated within each cohort and standardised relative to HC to produce cohort-specific healthy-control-referenced z-score matrices. Endotype discovery was performed exclusively in SLE samples using the aggregate-level matrix as clustering input. The k = 2 solution was retained as the working endotype configuration based on aggregate-level stability assessments (Figure S2).

### 2.3. Candidate Source Pool, Panel Compression, and Compact Score Derivation

Panel derivation was constrained to a locked biologically informed source pool derived from the whole-blood discovery stage (6233 genes) and filtered by cross-cohort directional consistency. Candidate modules retained biological provenance labels (interferon, lymphoid B/T/NK, myeloid/monocyte, neutrophil-inflammatory) to preserve interpretability. Redundancy control used pairwise gene-gene correlation with greedy pruning. Stepwise compression through locked intermediate shortlists (12, 10, 8 genes) followed by leave-one-gene-out analysis yielded two locked 6-gene candidates: pre6-any (*MMP9*, *MYL9*, *MME*, *HAL*, *CTLA4*, *CD40LG*) and pre6-balanced (*MMP9*, *MYL9*, *HAL*, *CTLA4*, *CD40LG*, *VPREB3*). For score construction, each locked gene was z-standardised across samples within each cohort, and predefined direction coefficients were applied so that higher sign-aligned values represented the myeloid/neutrophil-inflammatory side of the axis. For the locked pre6-balanced panel, *MMP9*, *MYL9* and *HAL* were positively aligned, whereas *CTLA4*, *CD40LG* and *VPREB3* were negatively aligned. The compact score was calculated as the unweighted arithmetic mean of the six sign-aligned gene-level z-scores. The transparent derivation workflow is summarised in Figure S3 and Methods S1.

### 2.4. Public Validation and Final Panel Locking

GSE138458 served as the prespecified primary validation cohort. At the architecture level, this cohort comprised 336 samples, including 312 SLE and 24 HC samples. The locked primary-validation compact-score analysis used an outlier-removed sample-level subset of 330 samples, including 307 SLE and 23 HC samples; within the SLE subset, the activity-related comparison included 156 high-activity and 151 low-activity samples. Six GSE138458 samples (1 HC and 5 SLE) had been designated as outliers in the source processing record and were excluded prior to normalisation; therefore, outlier removal preceded compact-score calculation and endpoint comparisons. Extended validation was performed in GSE65391 (main extended-validation adjudication cohort, one-subject-one-visit design), GSE110685 (cross-platform whole-blood RNA-seq cohort), and GSE61635 (supportive molecular cohort only; reliable sample-level activity annotations could not be restored, so it was excluded from principal final locking). Final panel locking was based on integrated public-stage validation evidence rather than discovery-stage performance alone. For the major extended-validation cohorts, retained endpoint domains were summarised as locked cohort-level adjudication outputs (total_score/rank) without additional post hoc reweighting after final locking. Additional detail is provided in Tables S2 and S3, and Methods S1. A post hoc sensitivity analysis rerunning the GSE138458 primary-validation workflow using all 336 architecture-level samples was performed after panel locking and did not participate in the locking decision or alter the final locked panel definition.

### 2.5. External Immune-State Comparison in GSE224705

After the final panel had been locked, the locked pre6-balanced compact score was applied to the independent longitudinal SLE/LN transcriptomic cohort GSE224705 as an external immune-state comparison. This analysis was performed without model refitting, threshold optimisation, or gene-panel modification. Predefined IFN, neutrophil/myeloid, and lymphoid T/B-cell signature scores were calculated using prespecified marker sets. The objective was to evaluate whether the locked compact score simply reproduced a canonical IFN-high/low classification or instead captured an immune-state axis more closely aligned with myeloid–lymphoid variation. Compact-score states and IFN-high/low states were defined using median splits for descriptive overlap analysis. Sample-level and patient-level sensitivity analyses were performed to assess robustness. These analyses were not used for panel derivation, public-stage adjudication, final panel selection, or local validation. Additional methodological detail is provided in Methods S3.

### 2.6. Single-Cell Biological Anchoring

Single-cell transcriptomic analysis was performed exclusively for biological anchoring and was not used for bulk discovery, public validation, or panel refinement [8,9]. GSE135779 served as the formal anchoring dataset. For each annotated cell type, panel-level scores were summarised within SLE and HC groups; the cell-type-specific panel shift was defined as Δscore = mean(SLE) − mean(HC). No cell-level inferential testing was applied. Anchoring results are presented descriptively in the Section 3. Methods S2 provides additional detail.

### 2.7. Local Validation Cohorts

The local PBMC validation cohort comprised 30 HC and 60 SLE patients sampled under a standardised clinical collection framework, with demographic, disease activity, serological, and treatment data recorded at PBMC sampling (Table 1). An expanded, additional local whole-blood RT-qPCR validation set included 30 HC and 60 patients with SLE. This set was used for compartment-matched orthogonal validation of the locked score, without gene replacement, model refitting, or threshold optimisation. Participants were enrolled under the same predefined inclusion criteria and were independent of the PBMC cohort, with no overlapping participants. Local RT-qPCR validation used the final locked 6-gene panel (*MMP9*, *MYL9*, *HAL*, *CTLA4*, *CD40LG*, *VPREB3*) in PBMC samples. The compact score was calculated using the same sign-aligned z-score averaging rule as in public validation. Flow-cytometric anchoring correlated the score with prespecified monocyte/myeloid readouts (classical, intermediate, nonclassical, and CD169-positive monocyte proportions) using Spearman correlation.

For the local PBMC RT-qPCR validation, PBMCs were isolated from EDTA-anticoagulated peripheral blood using human peripheral blood lymphocyte separation medium (Tianjin Haoyang Biological Manufacture Co., Ltd., Tianjin, China; Cat. No. LTS1077) according to the manufacturer’s instructions. Total RNA from PBMCs was extracted using TRIzol reagent (Life Technologies, Carlsbad, CA, USA; Cat. No. 15596018CN). Reverse transcription was performed using PrimeScript™ RT reagent Kit with gDNA Eraser (Takara Bio Inc., Shiga, Japan; Cat. No. RR047A). Quantitative PCR was performed using NovoStart SYBR qPCR SuperMix Plus (Novoprotein Scientific Inc., Suzhou, China; Cat. No. E096-01B) on a PIKOREAL 96 real-time PCR system (Thermo Fisher Scientific, Waltham, MA, USA). PikoReal Software v2.1 was used for qPCR data acquisition and analysis. β-actin was used as the internal reference gene, relative expression was calculated using the 2−ΔΔCt method, and each reaction was performed in duplicate technical wells. Primer sequences are available from the corresponding author upon reasonable request.

For the expanded whole-blood RT-qPCR validation set, total RNA was extracted from EDTA-anticoagulated whole blood using RNAprep Pure Hi-Blood Total RNA Kit (Tiangen Biotech Co., Ltd., Beijing, China; Cat. No. DP443). The reverse transcription kit, qPCR reagents, instrument, internal reference gene, primer sequences, and relative expression calculation method were the same as those used for the PBMC RT-qPCR validation.

Flow cytometry was performed on whole-blood samples after red blood cell lysis. Cells were stained using a service-provider monocyte antibody panel including CD14, CD16, and CD169/SIGLEC1, together with viability staining, and incubated in the dark for 15 min. Classical monocytes were defined as CD14++CD16−, intermediate monocytes as CD14++CD16+, and nonclassical monocytes as CD14+CD16++. CD169-positive monocytes were quantified according to the predefined service-provider gating template. Flow-cytometric data were acquired on a CytoFLEX flow cytometer using CytExpert v2.4.0.28 (Beckman Coulter, Brea, CA, USA) and analysed using FlowJo v10.8.1 (BD Biosciences, Ashland, OR, USA). Because detailed antibody clone and catalogue information was not available in the archived author-side method record, flow-cytometric findings were interpreted as supportive exploratory evidence.

### 2.8. Statistical Analysis

Compact-score comparisons used unpaired *t*-tests or Mann–Whitney U tests selected according to distributional characteristics. High disease activity was defined as SLEDAI > 6, and low/moderate activity was defined as SLEDAI ≤ 6. ROC analysis provided AUC estimates with 95% confidence intervals. Spearman correlation assessed score–clinical variable associations. Treatment-adjusted sensitivity analyses were adjusted for prespecified treatment exposure variables as reported in Tables S5 and S7. Benjamini–Hochberg false discovery rate correction was applied across PBMC-based statistical tests as a supplementary transparency check (Table S4). For the external GSE224705 immune-state comparison, Spearman correlations were calculated at both sample and patient levels, and Fisher’s exact test was used to evaluate overlap between compact-score states and IFN-high/low states. Exploratory response-stratified analyses in GSE224705 were reported descriptively in Supplementary Table S10 and were not used for treatment-response prediction or panel selection. Because the compact score was locked through public validation before local testing, local analyses were interpreted as prespecified supportive assessments. All tests were two-sided; *p* < 0.05 was considered statistically significant.

All statistical analyses and figure generation were performed in R v4.5.1. Key R packages included GEOquery v2.76.0, GSVA v2.2.0, ConsensusClusterPlus v1.72.0, limma v3.64.3, edgeR v4.6.3, Seurat v5.4.0, ComplexHeatmap v2.24.1, circlize v0.4.17, patchwork v1.3.2, ggpubr v0.6.3, data.table v1.18.2.1, tidyverse v2.0.0, pROC v1.19.0.1, ggplot2 v4.0.2, readxl v1.4.5, showtext v0.9-8, showtextdb v3.0, sysfonts v0.8.9, dplyr v1.2.0, RColorBrewer v1.1-3, scales v1.4.0, and tibble v3.3.1. Public code and additional analysis dependencies are available in the code repository cited in the Data Availability Statement.

### 2.9. Post Hoc Benchmarking Analyses

Post hoc benchmarking analyses were performed to contextualise the locked compact score against established immune and clinical readouts. These analyses did not alter the locked final panel, score-construction formula, endpoint definitions, or public-stage adjudication framework. The locked compact score remained the pre6-balanced panel, calculated within each cohort by gene-wise standardisation across samples, locked sign alignment, and unweighted averaging of the six aligned values.

Benchmarking analyses included comparisons against standard clinical markers in the local PBMC cohort, a fixed 6-gene IFN score in GSE224705 (*IFI27*, *IFI44L*, *IFIT1*, *ISG15*, *MX1*, and *OAS1*), individual locked panel genes, and reduced myeloid- and lymphoid-component scores. These analyses were used only to assess robustness, interpretability, and non-redundancy relative to established readouts, and did not participate in panel selection, public-stage locking, threshold optimisation, or reverse refinement of the final score. No formal cross-validation procedure was applied during discovery-stage feature selection. Instead, overfitting risk was addressed by restricting the candidate source pool through cross-cohort directional consistency, locking the final panel through independent public-stage validation before local testing, and evaluating robustness through withheld local validation and post hoc reduced-model benchmarking.

### 2.10. Ethics Statement

The local validation samples were collected under an approved SLE clinical study protocol. The study was approved by the Medical Ethics Committee of the First Affiliated Hospital of Anhui University of Chinese Medicine (approval No. 2024AH-08; approved 31 January 2024; validity period 31 January 2024 to 31 January 2026). Participants in the local PBMC validation cohort and the expanded whole-blood RT-qPCR validation set were prospectively recruited between 1 February 2024 and 30 January 2025 at the First Affiliated Hospital of Anhui University of Chinese Medicine. All participants provided written informed consent before sample collection. Publicly available transcriptomic datasets were obtained from GEO, BioStudies, and ArrayExpress; the authors had no access to information that could directly identify individual participants in these public datasets.

## 3. Results

### 3.1. Study Architecture and Staged Analytical Framework

The study was organised as a staged framework integrating public transcriptomic discovery, independent public validation, single-cell biological anchoring, and local orthogonal validation (Figure 1). Three discovery cohorts, one primary validation cohort, three extended validation cohorts, and two single-cell reference/resource datasets comprised the public resource set; a reserve cohort was listed separately. This architecture maintained clear separation between discovery, public-stage adjudication, biological anchoring, and local validation, preventing implicit overlap between feature selection and downstream validation.

### 3.2. Discovery-Stage Whole-Blood Endotypes Suggest a Working Myeloid–Lymphoid Axis

**Figure 2:**
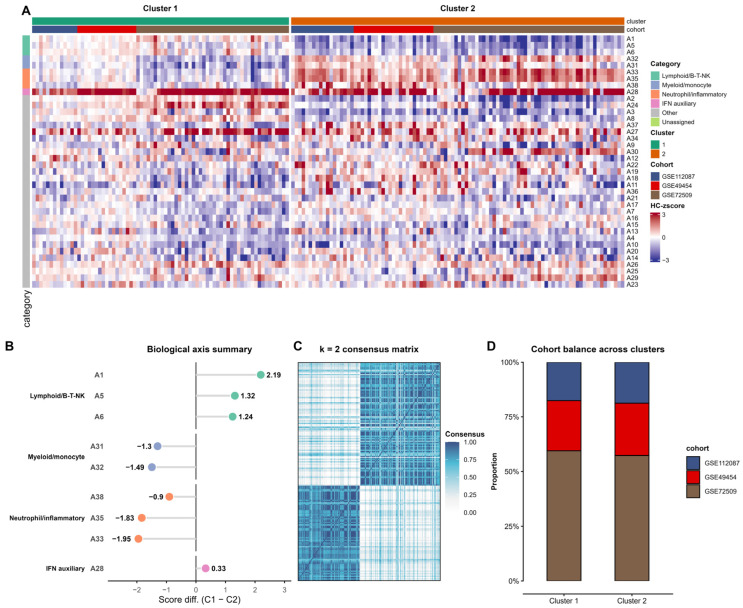
Discovery-stage whole-blood endotypes, biological axis, and stability support in SLE. (**A**) Heatmap of the BloodGen3 aggregate-level healthy-control-referenced z-score matrix restricted to discovery-stage SLE samples (*n* = 170), drawn from the final discovery analysis set of 233 samples, including 170 SLE and 63 HC. Samples were ordered by the k = 2 working solution; cluster 1 is shown first and cluster 2 s. Top annotations indicate cluster assignment and cohort origin; side annotations indicate the curated biological category. (**B**) Summary plot of representative aggregate-level differences between the two discovery working clusters. Positive values indicate relatively higher aggregate scores in cluster 1; negative values indicate relatively higher aggregate scores in cluster 2. Interferon-related signals are shown as an auxiliary interpretive layer only. (**C**) Consensus matrix for the k = 2 working solution, showing the block structure supporting the retained two-cluster configuration. (**D**) Cohort composition across the two discovery clusters, showing that the k = 2 solution was not driven by a single discovery cohort. Abbreviations: HC, healthy control; IFN, interferon; SLE, systemic lupus erythematosus.

Transcriptomic stratification of discovery-stage SLE samples (*n* = 170), drawn from a final discovery analysis set of 233 samples including 170 SLE and 63 HC, identified two working whole-blood endotypes (cluster 1, *n* = 74; cluster 2, *n* = 96) using the aggregate-level BloodGen3 healthy-control-referenced matrix (Figure 2A). Cluster 1 showed relatively higher lymphoid-related activity; cluster 2 showed relative enrichment of myeloid/monocyte and neutrophil/inflammatory programmes (Figure 2B). Interferon-related signals were detectable but did not provide formal support for interferon as the principal organising axis. The discovery results were therefore interpreted conservatively as reflecting a lymphoid versus myeloid/neutrophil-inflammatory polarisation rather than an interferon-defined split. The k = 2 consensus matrix showed clear block structure supporting the retained two-cluster configuration (Figure 2C). The cohort composition across the two clusters confirmed balanced representation of all three discovery cohorts, indicating that the k = 2 solution was not driven by any single contributing cohort (Figure 2D). Expanded stability evidence is provided in Figure S2.

### 3.3. Public-Stage Evidence Locks pre6-Balanced as the Final Compact Panel

**Figure 3:**
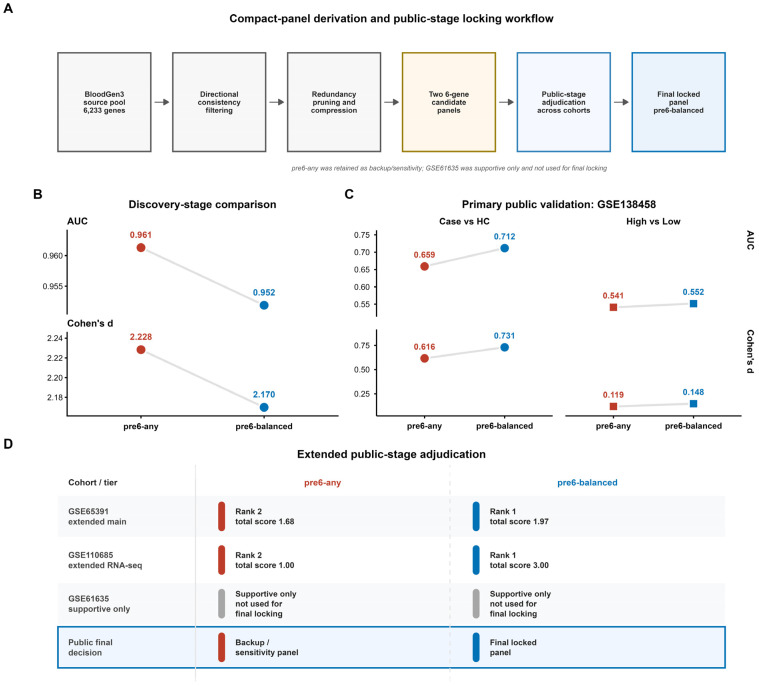
Compact-panel derivation, public-stage comparison, and final locking of the compact score panel. (**A**) Schematic summary of the compact-panel derivation and public-stage locking workflow. A BloodGen3-derived source pool of 6233 genes was filtered by cross-cohort directional consistency and redundancy pruning, yielding two 6-gene candidate panels for public-stage adjudication. (**B**) Discovery-stage comparison of pre6-any and pre6-balanced in pooled discovery whole-blood samples. Pre6-any showed a marginal discovery-stage advantage (AUC 0.961 vs. 0.952; Cohen’s d 2.228 vs. 2.170). (**C**) Primary public validation in the predefined outlier-removed GSE138458 subset (*n* = 330). Pre6-balanced showed stronger performance than pre6-any in case-versus-HC discrimination (AUC 0.712 vs. 0.659; Cohen’s d 0.731 vs. 0.616) and weakly favoured pre6-balanced in activity-related comparisons. A post hoc all-336 sensitivity analysis is reported in Table S2 and was not used for panel locking. (**D**) Extended public-stage adjudication across GSE65391 and GSE110685, both of which ranked pre6-balanced above pre6-any. In GSE65391, locked cohort-level aggregation ranked pre6-balanced first (rank 1, total_score 1.97 vs. rank 2, total_score 1.68); in GSE110685, pre6-balanced was again ranked first (rank 1, total_score 3.00 vs. rank 2, total_score 1.00). GSE61635 was retained as supportive molecular evidence only and was not used for final locking. Pre6-balanced was locked as the final main panel after public-stage adjudication before local validation; pre6-any was retained as a backup/sensitivity panel. Abbreviations: AUC, area under the receiver operating characteristic curve; HC, healthy control; RNA-seq, RNA sequencing; SLE, systemic lupus erythematosus.

Constrained panel compression yielded two 6-gene candidates. The compact-panel derivation and public-stage locking workflow is summarised schematically in Figure 3A. In pooled discovery, pre6-any showed a marginal advantage over pre6-balanced in discrimination (AUC 0.961 vs. 0.952) and effect-size performance (Cohen’s d 2.228 vs. 2.170) (Figure 3B). However, final adjudication was based on staged public validation to identify the most transportable compact score. In the predefined outlier-removed primary-validation subset of GSE138458 (*n* = 330; 307 SLE, 23 HC), pre6-balanced showed stronger performance than pre6-any in case-versus-HC discrimination (AUC 0.712 vs. 0.659; Cohen’s d 0.731 vs. 0.616) and weakly favoured pre6-balanced in activity-related comparisons (both panels weak; Figure 3C). A post hoc sensitivity analysis including all 336 architecture-level samples of GSE138458 did not fully maintain this preference: the case–control endpoint favoured pre6-any (AUC 0.684 vs. 0.627), whereas the activity-related comparison remained weak, with only slight residual directional support for pre6-balanced (Table S2). The final panel decision was therefore based on integrated public-stage evidence rather than on the GSE138458 primary-validation analysis alone. Extended public-stage adjudication across GSE65391 and GSE110685 both ranked pre6-balanced above pre6-any: in GSE65391, locked cohort-level aggregation ranked pre6-balanced first (rank 1, total_score 1.97 vs. rank 2, total_score 1.68); in GSE110685, pre6-balanced was again ranked first (rank 1, total_score 3.00 vs. rank 2, total_score 1.00) (Figure 3D). GSE61635 provided supportive SLE-versus-HC molecular evidence only and was excluded from principal adjudication. Taken together, the integrated public-stage adjudication supported formal locking of pre6-balanced (*MMP9*, *MYL9*, *HAL*, *CTLA4*, *CD40LG*, *VPREB3*) as the final main panel before any local validation; pre6-any was retained as a sensitivity/backup panel (Tables S2 and S3).

### 3.4. Local PBMC Validation Supports Disease-Context Relevance of the Locked Compact Score

The local PBMC validation cohort included 30 HC and 60 SLE patients (Table 1). Baseline age, sex, and BMI were broadly comparable between groups. As expected, the SLE group showed lower C3 and C4 levels and higher anti-dsDNA titres, while routine haematological indices were similar. Treatment exposure differed substantially between groups.

**Figure 4:**
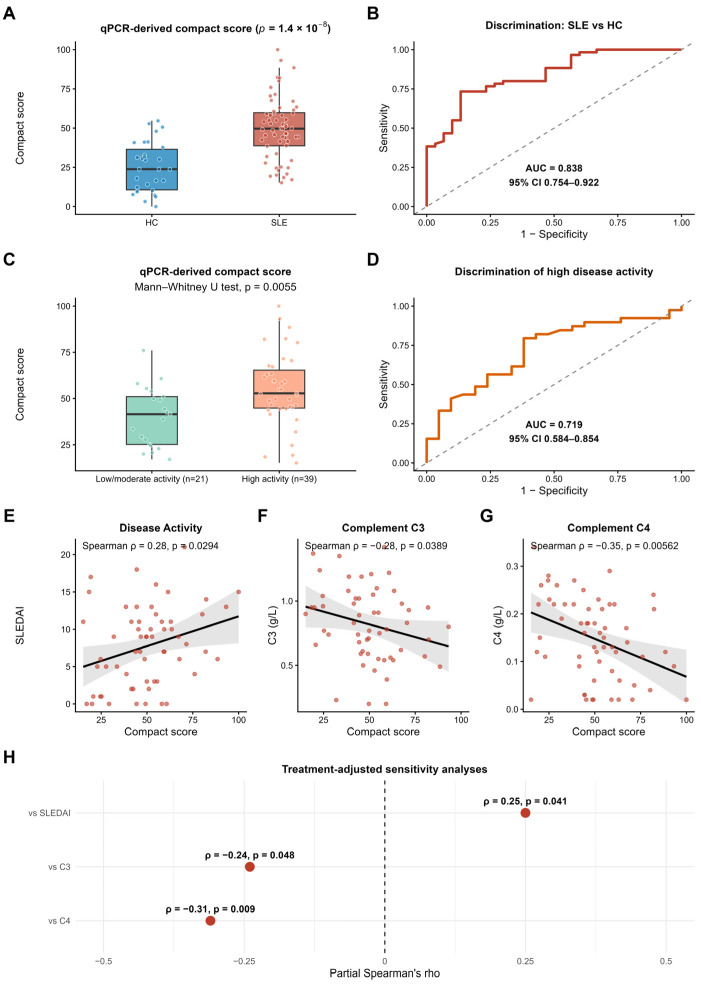
Local PBMC validation of the locked compact score. (**A**) Compact score comparison between HC and SLE (Student’s *t*-test, *p* = 1.4 × 10^−8^; Cohen’s d = 1.62). (**B**) ROC curve for SLE-versus-HC discrimination (AUC 0.838, 95% CI 0.754–0.922). (**C**) Compact score by disease activity subgroup (High vs. Low/Moderate; Mann–Whitney U test, *p* = 0.0055). (**D**) ROC curve for high versus low/moderate disease activity discrimination (AUC 0.719, 95% CI 0.584–0.854). (**E**–**G**) Spearman correlations between the compact score and SLEDAI (ρ = 0.28, *p* = 0.029), C3 (ρ = −0.28, *p* = 0.039), and C4 (ρ = −0.35, *p* = 0.0056) within SLE patients. (**H**) Treatment-adjusted sensitivity analyses: partial Spearman correlations for SLEDAI (ρ = 0.25, *p* = 0.041), C3 (ρ = −0.24, *p* = 0.048), and C4 (ρ = −0.31, *p* = 0.009). Abbreviations: AUC, area under the receiver operating characteristic curve; CI, confidence interval; HC, healthy control; PBMC, peripheral blood mononuclear cell; SLE, systemic lupus erythematosus; SLEDAI, Systemic Lupus Erythematosus Disease Activity Index.

The locked compact score was significantly elevated in SLE relative to HC (Student’s *t*-test, *p* = 1.4 × 10^−8^; Cohen’s d = 1.62) (Figure 4A) with ROC-supported discrimination (AUC 0.838, 95% CI 0.754–0.922) (Figure 4B). Within the SLE group, the score was significantly higher in patients with high disease activity than in those with low/moderate activity (Mann–Whitney U test, *p* = 0.0055; AUC 0.719, 95% CI 0.584–0.854) (Figure 4C,D). The locked score correlated positively with SLEDAI (ρ = 0.28, *p* = 0.029) and inversely with C3 (ρ = −0.28, *p* = 0.039) and C4 (ρ = −0.35, *p* = 0.0056) (Figure 4E–G). These associations remained significant after treatment adjustment (SLEDAI: ρ = 0.25, *p* = 0.041; C3: ρ = −0.24, *p* = 0.048; C4: ρ = −0.31, *p* = 0.009) (Figure 4H; Table S5). All six locked panel genes showed directionally concordant between-group differences in local PBMC samples (Figure S1).

### 3.5. Biological Anchoring and Expanded Whole-Blood Validation Support the Locked Score

**Figure 5:**
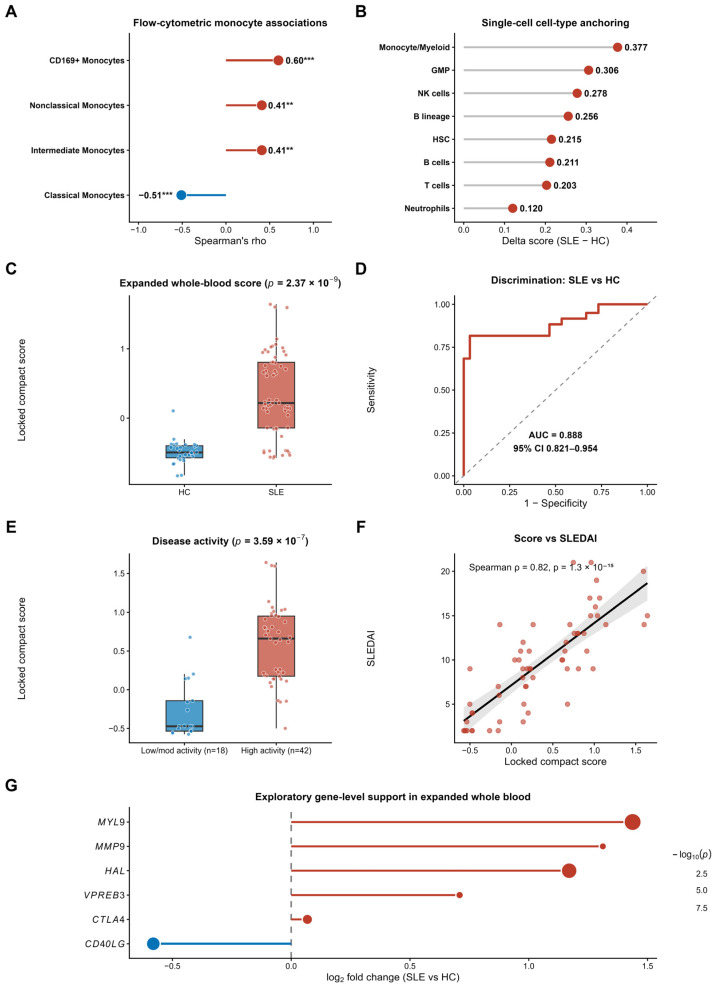
Biological anchoring and expanded whole-blood validation of the locked compact score. (**A**) Spearman correlations between the locked compact score and prespecified monocyte subpopulations in the local PBMC cohort: CD169-positive monocytes (ρ = 0.60, *p* = 4.1 × 10^−7^), nonclassical monocytes (ρ = 0.41, *p* = 0.0013), intermediate monocytes (ρ = 0.41, *p* = 0.0024), and classical monocytes (ρ = −0.51, *p* = 3.2 × 10^−5^). (**B**) Descriptive single-cell cell-type anchoring using GSE135779. For each annotated cell type, the panel shift is shown as Δscore = mean(panel score in SLE) − mean(panel score in HC). This analysis was used only for biological anchoring and not for panel selection or refinement. (**C**) Compact score in HC versus SLE in the expanded additional local whole-blood RT-qPCR validation set (30 HC and 60 SLE; Mann–Whitney U test, *p* = 2.37 × 10^−9^). (**D**) ROC curve for SLE-versus-HC discrimination in the expanded whole-blood validation set (AUC = 0.888, 95% CI 0.821–0.954). (**E**) Compact score by disease activity subgroup in the expanded whole-blood validation set (low/moderate activity *n* = 18; high activity *n* = 42; Mann–Whitney U test, *p* = 3.59 × 10^−7^). (**F**) Spearman correlation between the whole-blood compact score and SLEDAI within patients with SLE (ρ = 0.819, *p* = 1.25 × 10^−15^). (**G**) Condensed gene-level summary in the expanded whole-blood cohort, showing gene-level direction and statistical strength for the locked 6-gene panel. Full gene-level RT-qPCR distributions are shown in Figure S5. Significance symbols: ** *p* < 0.01; *** *p* < 0.001. Gene-level results were interpreted as exploratory support, whereas the primary whole-blood validation was based on the locked composite compact score. Additional robustness analyses for the expanded whole-blood validation set, including leave-one-out sample analysis, bootstrap resampling and LOGO analysis, are provided in Table S7. In LOGO analysis, the score was recalculated using the locked sign-aligned z-score averaging rule for the remaining five genes without model refitting. Abbreviations: AUC, area under the receiver operating characteristic curve; CI, confidence interval; HC, healthy control; PBMC, peripheral blood mononuclear cell; SLE, systemic lupus erythematosus; SLEDAI, Systemic Lupus Erythematosus Disease Activity Index.

The compact score correlated with prespecified monocyte/myeloid flow-cytometric readouts: CD169-positive monocyte (%) (ρ = 0.60, *p* = 4.1 × 10^−7^), nonclassical monocyte (%) (ρ = 0.41, *p* = 0.0013), intermediate monocyte (%) (ρ = 0.41, *p* = 0.0024), and classical monocyte (%) (ρ = −0.51, *p* = 3.2 × 10^−5^) (Figure 5A). These findings provided local immunophenotypic support for the association of the locked PBMC-derived score with a recognisable monocyte/myeloid activation context.

Single-cell anchoring using GSE135779 showed descriptively that the pre6-balanced panel shift (Δscore = mean SLE − mean HC) was largest in monocyte/myeloid (Δ = 0.377) and GMP (Δ = 0.306) compartments, with contributions from NK cells (Δ = 0.278), B lineage (Δ = 0.256), HSC (Δ = 0.215), B cells (Δ = 0.211), T cells (Δ = 0.203), and neutrophils (Δ = 0.120) (Figure 5B). This pattern was consistent with the discovery-stage whole-blood structure and supported the myeloid–lymphoid activity interpretation of the locked score. These cell-type shifts were interpreted descriptively as biological anchoring rather than independent inferential evidence.

In the expanded, additional whole-blood RT-qPCR validation set (30 HC and 60 SLE), the locked compact score was significantly higher in SLE than in HC (Mann–Whitney U test, *p* = 2.37 × 10^−9^) and showed strong ROC-supported SLE-versus-HC discrimination (AUC = 0.888, 95% CI 0.821–0.954) (Figure 5C,D). Within patients with SLE, the score was significantly higher in the high disease activity group than in the low/moderate activity group (*p* = 3.59 × 10^−7^; low/moderate *n* = 18, high *n* = 42; AUC = 0.918) and correlated strongly with SLEDAI (Spearman ρ = 0.819, *p* = 1.25 × 10^−15^) (Figure 5E,F). By contrast, the score did not correlate significantly with C3 in this expanded whole-blood set (ρ = −0.058, *p* = 0.657; Table S7). Gene-level direction and statistical strength for the locked 6-gene panel in the expanded whole-blood cohort are summarised in Figure 5G; full gene-level RT-qPCR distributions are shown in Figure S5. Gene-level results were interpreted as exploratory support, whereas the primary whole-blood validation was based on the locked composite compact score. Additional robustness analyses showed that the whole-blood score–SLEDAI association was stable in leave-one-out sample analysis (ρ range, 0.811–0.842) and bootstrap resampling (ρ = 0.819, 95% CI 0.704–0.890). Bootstrap analyses also supported stable SLE-versus-HC discrimination (AUC = 0.888, 95% CI 0.813–0.951) and high-versus-low/moderate activity discrimination (AUC = 0.918, 95% CI 0.831–0.980). LOGO analysis indicated that the composite score was not dominated by a single locked gene (Table S7). These findings support compartment-matched reproducibility of the locked compact score and provide additional activity-related support in whole blood.

### 3.6. External GSE224705 Analysis Distinguishes the Compact Score from IFN-High/Low Classification

**Figure 6:**
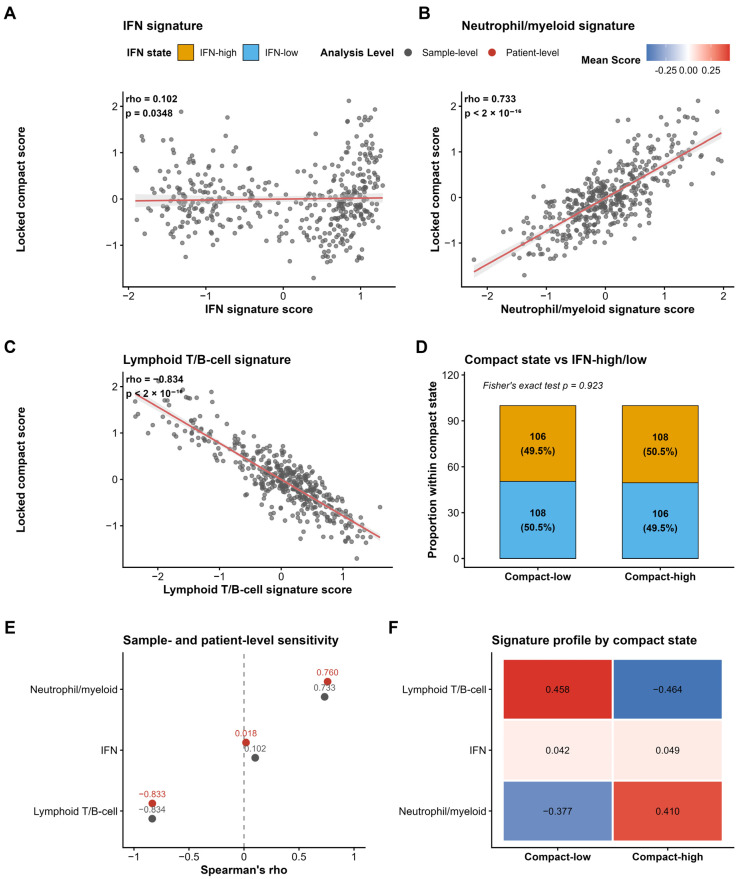
External immune-state comparison of the locked compact score in GSE224705. The locked pre6-balanced compact score was applied to the independent longitudinal SLE/LN transcriptomic cohort GSE224705 to evaluate whether it simply reproduced canonical IFN-high/low classification. (**A**) Sample-level correlation between the locked compact score and an IFN signature score (Spearman ρ = 0.102, *p* = 0.0349). (**B**) Sample-level correlation between the locked compact score and a neutrophil/myeloid signature score (Spearman ρ = 0.733, *p* < 2 × 10^−16^). (**C**) Sample-level correlation between the locked compact score and a lymphoid T/B-cell signature score (Spearman ρ = −0.834, *p* < 2 × 10^−16^). Sample-level Spearman correlation coefficients and *p* values are shown in panels (**A**–**C**); corresponding patient-level sensitivity results are summarised in panel (**E**). (**D**) Distribution of IFN-high and IFN-low samples within median-defined compact-score states. Compact-high represents the myeloid/neutrophil-inflammatory side of the locked score; compact-low represents the lymphoid side/lower-score state. Near-even IFN-high/low distribution was observed in both compact states (Fisher’s exact test *p* = 0.923). (**E**) Sample-level and patient-level sensitivity correlation summary for the relationship between the compact score and the three immune signature axes. (**F**) Signature-profile heatmap comparing compact-low and compact-high states. IFN signals showed no clear enrichment between compact states; neutrophil/myeloid signals were higher in the compact-high state; lymphoid T/B-cell signals were higher in the compact-low state. These analyses were performed as an external immune-state comparison and were not used for panel derivation, model refitting, threshold optimisation, or final panel selection. Overlap-removed sensitivity analyses are provided in Table S9 and were performed without modifying the locked compact score. Abbreviations: IFN, interferon; SLE, systemic lupus erythematosus.

After final panel locking, the locked pre6-balanced compact score was applied to the independent longitudinal SLE/LN transcriptomic cohort GSE224705 as an external immune-state comparison, without model refitting, threshold optimisation, or gene-panel modification. The compact score showed only a weak association with a predefined IFN signature at the sample level (Spearman ρ = 0.102, *p* = 0.0349) and no significant association at the patient level (ρ = 0.0185, *p* = 0.814) (Figure 6A). By contrast, the compact score aligned strongly with a neutrophil/myeloid signature (sample-level ρ = 0.733, *p* < 2 × 10^−16^; patient-level ρ = 0.760, *p* < 2 × 10^−16^) (Figure 6B) and showed strong inverse association with a lymphoid T/B-cell signature (sample-level ρ = −0.834, *p* < 2 × 10^−16^; patient-level ρ = −0.833, *p* < 2 × 10^−16^) (Figure 6C). When compact-score states were defined by median split, IFN-high and IFN-low samples were distributed near-evenly across compact-high (myeloid-side; IFN-high *n* = 108, IFN-low *n* = 106) and compact-low (lymphoid-side; IFN-high *n* = 106, IFN-low *n* = 108) states (Fisher’s exact test *p* = 0.923) (Figure 6D). Sample-level and patient-level sensitivity analyses showed consistent correlation patterns for the signature axes (Figure 6E). The signature-profile heatmap showed that IFN signals were distributed without clear enrichment between compact states, whereas neutrophil/myeloid signals were higher in the compact-high state and lymphoid T/B-cell signals were higher in the compact-low state (Figure 6F). Full signature-level results are reported in Table S9. Together, these analyses supported the interpretation that the locked compact score captured a myeloid–lymphoid activity axis that was not reducible to IFN-high/low classification in this external cohort. The correlation pattern remained directionally consistent after removing genes overlapping with the locked compact panel from the reference immune signatures (Table S9). GSE224705 was used solely for external immune-state comparison and was not used for panel derivation, model refitting, threshold optimisation, or final panel selection.

### 3.7. Post Hoc Benchmarking Against IFN, Clinical-Marker, and Reduced-Score Readouts

To contextualise the locked compact score relative to established immune and clinical readouts, we performed post hoc benchmarking analyses without altering the locked panel or score formula (Tables S11 and S12). In the local PBMC cohort, the compact score showed activity-related discrimination within the range of complement markers (compact score AUC = 0.719; C3 AUC = 0.754; C4 AUC = 0.731), whereas anti-dsDNA showed limited discrimination in this dataset (AUC = 0.503). In GSE224705, the fixed 6-gene IFN score showed stronger association with SLEDAI-defined disease activity and stronger discrimination of high-versus-low/moderate activity and SLE/LN-versus-healthy-control status than the locked compact score. However, the compact score was only weakly correlated with the IFN score (ρ = 0.102, *p* = 3.483 × 10^−2^), supporting complementary rather than redundant biological information. Single-gene and reduced-score benchmarking showed that some individual or reduced readouts could approach the full compact score in local validation tasks. In the expanded whole-blood validation set, the full compact score retained the highest activity-related AUC and strongest SLEDAI correlation among the tested readouts, although *MMP9* and the myeloid half-score approached its performance. These post hoc analyses did not alter the locked-panel definition and were not used for gene re-selection.

## 4. Discussion

In this study, we established a staged framework for peripheral-blood transcriptomic stratification in SLE in which whole-blood discovery, public-stage panel adjudication, single-cell biological anchoring, and local orthogonal validation served distinct and non-interchangeable roles. We identified two working whole-blood endotypes characterised by lymphoid versus myeloid/neutrophil-inflammatory polarisation and translated that structure into a compact 6-gene score locked through public validation before any local testing [3,4,5]. The expanded, additional whole-blood RT-qPCR validation set strengthened the validation structure by providing compartment-matched support, including strong activity-related associations with SLEDAI, for a score originally derived and adjudicated in public whole-blood cohorts.

The panel-selection process illustrates why staged public locking matters. In pooled discovery data, pre6-any showed a marginal advantage over pre6-balanced (AUC 0.961 vs. 0.952; Cohen’s d 2.228 vs. 2.170). Had panel selection been driven by discovery-stage optimisation alone, pre6-any might have been carried forward as the leading signature. However, when evaluated in the predefined outlier-removed primary-validation subset of GSE138458 and across major extended-validation cohorts, the integrated public-stage adjudication favoured pre6-balanced. A post hoc all-336 sensitivity analysis in GSE138458 showed that this primary-cohort preference was not fully stable to inclusion of the six source-designated outliers, with reversal of the case–control endpoint and only weakly similar activity-related direction. Locking pre6-balanced, therefore, reflected integrated public-stage adjudication rather than a single-cohort or single-endpoint conclusion, and the final panel decision should be interpreted accordingly rather than as uniquely driven by the GSE138458 primary-validation analysis. This underscores the importance of prioritising multi-cohort external evidence when deriving compact molecular classifiers intended for downstream translational use [4,6].

A key biological interpretation is that the observed whole-blood structure reflects a myeloid–lymphoid activity axis rather than a formally interferon-defined split. This requires careful qualification. Blood transcriptomic studies in SLE have long identified type I interferon-inducible and granulopoiesis/neutrophil-associated signatures as major peripheral-blood immune programmes [13,14]. The framework rests on aggregate-level BloodGen3 profiling, and different analytical approaches may yield different interpretations. Formal testing confirmed a statistically detectable interferon difference between discovery clusters, but this was numerically smaller than myeloid/monocyte and neutrophil/inflammatory contrasts. IFN-high and myeloid-high immune states are partially co-expressed in SLE, and the present framework does not argue that interferon signals are absent or unimportant—rather, they did not emerge as the dominant axis of unsupervised endotype separation [15,16,17]. The myeloid–lymphoid interpretation was supported descriptively across validation layers: the locked score mapped most prominently to monocyte/myeloid and progenitor-like compartments in single-cell anchoring and showed concordant relationships with monocyte-related immunophenotypes in the local PBMC cohort [8,18]. This interpretation was further supported by the external GSE224705 immune-state comparison, in which the locked compact score showed only weak association with an IFN signature and near-even overlap with IFN-high/low states, while aligning strongly with neutrophil/myeloid and inversely with lymphoid T/B-cell signatures. This analysis was intended to contextualise the immune-state meaning of the locked score and should not be interpreted as treatment-response prediction. Post hoc benchmarking in GSE224705 indicated that a fixed 6-gene interferon score (*IFI27*, *IFI44L*, *IFIT1*, *ISG15*, *MX1*, *OAS1*) showed stronger association with SLEDAI-defined disease activity (ρ = 0.417) and stronger SLE/LN-versus-healthy-control discrimination (AUC = 0.783) than the locked compact score in this cohort; these results are reported transparently in Supplementary Table S11. The two scores were only weakly correlated (ρ = 0.102), supporting complementary rather than redundant biological information. The compact score should therefore not be interpreted as a replacement for canonical interferon activity measures; its value lies in capturing a myeloid–lymphoid activity axis that is largely non-redundant with IFN-high/low classification.

The local validation results support the biological and disease-context relevance of the locked compact score. At the PBMC level, the score discriminated SLE from HC, separated high from low/moderate disease activity, and showed coherent associations with SLEDAI and complement consumption—associations that remained significant after treatment adjustment. It also correlated with monocyte-related immunophenotypes, including the CD169-positive monocyte compartment [19,20,21]. These findings do not establish clinical utility or suggest the score should replace standard serological indices. Post hoc benchmarking against standard clinical markers in the local PBMC cohort (Supplementary Table S11) showed that the compact score (SLEDAI ρ = 0.281, high versus low/moderate activity AUC = 0.719) captured activity-related information numerically within a comparable range to C4 (ρ = −0.422, AUC = 0.731) and C3 (AUC = 0.754), whereas anti-dsDNA showed no meaningful activity-related association in this setting (ρ = 0.008, AUC = 0.503). These results do not establish clinical superiority or equivalence and are reported as post hoc contextualisation only. Because the compact score is calculated using cohort-wise z-standardisation, clinically interpretable individual-level thresholds cannot be derived from the current data. Future clinical translation would require prospective validation with fixed calibration references, formal evaluation of additive predictive value over standard clinical parameters, and assessment of score behaviour across distinct disease manifestations. The expanded whole-blood RT-qPCR validation set provided compartment-matched corroboration: the locked score showed strong SLE-versus-HC discrimination and a strong positive association with SLEDAI across the expanded set. Although the C3 association observed in the PBMC layer was not reproduced in the expanded whole-blood set, the activity-related results support the view that the compact score captures a disease-context signal in the whole-blood compartment. The difference in activity-related discrimination between the PBMC layer (AUC = 0.719) and the expanded whole-blood validation set (AUC = 0.918) warrants comment. Several complementary explanations are plausible. First, the compact score was derived from whole-blood discovery cohorts, and the whole-blood compartment represents the discovery-matched measurement context; PBMC sampling introduces a cell-separation step that depletes granulocytes and alters the relative proportions of myeloid cell populations, potentially attenuating the myeloid-dominant signal. Second, the myeloid-component genes in the locked panel—particularly *MMP9* and *MYL9*—are highly expressed in granulocytes and monocytes, which are substantially enriched in whole blood relative to PBMC fractions. Third, RT-qPCR in the whole-blood compartment may capture a broader myeloid transcriptional signal. Together, these considerations suggest that the performance difference is plausibly related to compartment-specific biological and technical factors; however, cohort-specific effects cannot be fully excluded and are acknowledged as a limitation. In the expanded whole-blood cohort, several individual genes showed borderline or non-significant between-group differences, whereas the locked composite score showed stronger activity-related performance. This pattern should not be interpreted as local re-selection evidence for individual genes. Rather, the score was designed as a predefined sign-aligned multi-gene axis, and its interpretation depends on the composite signal rather than on each gene reaching significance in every validation compartment. Post hoc benchmarking against individual panel genes and reduced composite scores (Supplementary Table S12) showed that the strongest simplified readouts approached the performance of the full compact score in some local validation tasks. In the expanded whole-blood cohort, the full compact score retained the highest activity-related AUC and strongest SLEDAI correlation among all tested readouts (AUC = 0.918, ρ = 0.819), although *MMP9* alone (AUC = 0.899, ρ = 0.617) and the myeloid half-score (AUC = 0.907, ρ = 0.711) approached its performance, indicating that the myeloid component contributes substantially to the whole-blood activity signal. In the PBMC compartment, individual gene performance was more heterogeneous, and no single gene or reduced score uniformly reproduced the cross-layer rationale of the full locked panel. These findings indicate that the full score provides an interpretable composite representation of the pre-specified myeloid–lymphoid axis. Accordingly, the added value of the full composite score should not be interpreted as uniform predictive superiority over every simplified or single-gene readout in all compartments. Rather, its value lies in providing a pre-specified, locked, and biologically interpretable composite representation of the myeloid–lymphoid axis across validation layers. Importantly, these post hoc results should not be interpreted as evidence for retrospective panel revision; the panel was locked through public-stage adjudication before any local testing. Robustness analyses further suggested that the whole-blood score was not driven by a single sample or by a single locked gene, although prospective validation remains necessary.

Several limitations should be acknowledged. First, public datasets differed in platform, sampling context, and phenotypic annotation depth, constraining clinical harmonisation across validation layers. Second, the PBMC cohort represents a different blood compartment from the whole-blood discovery setting, and included substantial treatment exposure; local PBMC findings should therefore be interpreted as biological corroboration rather than direct replication of the whole-blood discovery layer. Although treatment-adjusted sensitivity analyses confirmed that associations with SLEDAI and complement levels remained statistically significant after adjustment for prespecified treatment variables, residual confounding cannot be excluded. In particular, glucocorticoids and other immunosuppressive agents are known to modulate myeloid transcriptional programmes, and their influence on the compact score signal cannot be fully disentangled in the current cross-sectional design. Larger prospective studies with treatment-stratified designs will be required to clarify the relative contributions of disease activity and treatment exposure to the observed transcriptomic axis. Third, the single-cell layer was restricted to biological anchoring and cannot serve as an independent panel for adjudication. Fourth, serological correlations were not uniformly reproduced across compartments—the C3 association observed in the PBMC layer was not confirmed in the expanded whole-blood set, which underscores the compartment-specific nature of complement associations and the need for careful interpretation across different measurement contexts. Fifth, the GSE224705 analysis was exploratory and external to the locking framework; response-stratified analyses by treatment stratum were underpowered and did not support treatment-response prediction or drug-selection claims. Sixth, in GSE138458, the predefined primary-validation analysis used a source-designated outlier-removed subset (*n* = 330). A post hoc all-336 sensitivity analysis showed that the GSE138458 case–control preference for pre6-balanced was not maintained after including these six samples, although the activity-related comparison remained weakly directionally similar. This underscores that the final panel-locking decision should be interpreted as integrated public-stage evidence across multiple cohorts rather than reliance on a single primary-validation endpoint. Seventh, formal cross-validation procedures were not applied within the discovery-stage feature selection process; although the multi-cohort public-stage adjudication and withheld local validation were intended to mitigate overfitting risk, a degree of analytical flexibility inherent in the multi-step compression workflow cannot be entirely eliminated. Finally, although the compact score showed coherent associations with disease activity and monocyte-related phenotypes, its value for longitudinal immune-state monitoring and prospective clinical stratification remains to be established. Because the compact score was calculated using cohort-wise standardisation, it should be interpreted as a research-grade relative activity score rather than a ready-to-use individual clinical assay. Future prospective studies should establish fixed calibration references and clinically interpretable cut-offs. In addition, although the framework integrates multi-layer transcriptomic, single-cell, and clinical information, it should not be interpreted as a formal systems-biology model in the strict sense. The study does not model dynamic feedback loops, regulatory networks, or mechanistic causal interactions. Rather, it represents a staged, systems-oriented biomarker-reduction framework that uses public validation and biological anchoring to derive a compact, interpretable transcriptomic readout.

## 5. Conclusions

We established a staged whole-blood transcriptomic framework for SLE and derived a compact myeloid–lymphoid activity score (pre6-balanced: *MMP9*, *MYL9*, *HAL*, *CTLA4*, *CD40LG*, *VPREB3*) supported by public validation, single-cell biological anchoring, and local orthogonal validation. Compartment-matched whole-blood RT-qPCR validation further supported score reproducibility and activity-related relevance in the same blood compartment as discovery, including a strong positive association with SLEDAI. However, the compact score should be interpreted as a cohort-normalised, research-grade relative activity readout rather than a calibrated clinical assay. The present study did not establish fixed calibration references, clinically interpretable thresholds, or decision rules for individual patient classification. In addition, although staged public validation, withheld local validation, bootstrap resampling, leave-one-out analysis, and reduced-model benchmarking mitigate overfitting concerns, formal cross-validation was not applied during discovery-stage feature selection. Post hoc benchmarking also showed that simplified or single-gene readouts may approach the full score in selected settings; therefore, the added value of the composite score should be interpreted primarily as a locked, biologically interpretable representation of a myeloid–lymphoid transcriptomic axis rather than as uniform predictive superiority over simpler alternatives. Further prospective, multicentre, treatment-aware studies with fixed calibration references are required to determine whether this score has value for longitudinal immune-state monitoring or prospective clinical stratification in SLE.

## Figures and Tables

**Table 1:** Clinical characteristics of the local PBMC validation cohort.

Characteristic	HC (*n* = 30)	SLE (*n* = 60)	*p* Value
Demographics			
Participants, *n*	30	60	—
Female sex, *n* (%)	22 (73.3%)	53 (88.3%)	0.072
Age, years	36.50 [27.25–41.00]	37.50 [29.00–43.00]	0.572
BMI, kg/m^2^	21.30 [20.10–24.00]	22.40 [19.60–24.80]	0.719
Disease activity and serology			
Disease duration, years	—	7.65 [4.92–12.80]	—
SLEDAI	—	7.50 [3.00–11.00]	—
C3, g/L	1.11 [0.97–1.29]	0.81 [0.60–1.02]	<0.001 *
C4, g/L	0.24 [0.18–0.30]	0.15 [0.09–0.22]	<0.001 *
anti-dsDNA, IU/mL	3.00 [2.00–3.50]	34.30 [20.80–71.70]	<0.001 *
Haematology			
WBC, ×10^9^/L	6.55 [5.67–7.11]	6.37 [5.15–7.40]	0.755
Lymphocyte, ×10^9^/L	1.79 [1.36–1.99]	1.56 [1.03–1.88]	0.058
Monocyte, ×10^9^/L	0.47 [0.39–0.60]	0.50 [0.38–0.57]	0.993
Neutrophil, ×10^9^/L	4.32 [3.19–4.78]	4.10 [3.07–5.00]	0.867
Platelet, ×10^9^/L	220.00 [195.00–271.00]	209.00 [169.00–243.25]	0.188
Treatment exposure			
Prednisone equivalent, mg/day	0.00 [0.00–0.00]	17.00 [7.75–29.25]	<0.001 *
Hydroxychloroquine use, *n* (%)	0 (0.0%)	52 (86.7%)	<0.001 *
MMF use, *n* (%)	0 (0.0%)	9 (15.0%)	0.027 *
AZA use, *n* (%)	0 (0.0%)	11 (18.3%)	0.014 *
Tacrolimus use, *n* (%)	0 (0.0%)	2 (3.3%)	0.551
Cyclophosphamide in past 3 months, *n* (%)	0 (0.0%)	1 (1.7%)	1.000
Belimumab current, *n* (%)	0 (0.0%)	10 (16.7%)	0.027 *
Major treatment change in prior 4 weeks, *n* (%)	0 (0.0%)	2 (3.3%)	0.551

Data are presented as median [interquartile range] for continuous variables and *n* (%) for categorical variables. *p*-values compare healthy controls and patients with SLE. Continuous variables were compared using the Mann–Whitney U test; categorical variables were compared using the chi-square test or Fisher’s exact test. * indicates *p* < 0.05. Abbreviations: AZA, azathioprine; BMI, body mass index; HC, healthy control; MMF, mycophenolate mofetil; PBMC, peripheral blood mononuclear cell; SLE, systemic lupus erythematosus; SLEDAI, Systemic Lupus Erythematosus Disease Activity Index; WBC, white blood cell count.

## Data Availability

Public datasets analysed in this study are available from the Gene Expression Omnibus and other public repositories under the accession numbers listed in Table S1. Processed data and source data supporting the analyses are available in Zenodo [22]. Local validation data are provided in de-identified, aggregated or processed form where applicable; additional participant-level data may be available from the corresponding author upon reasonable request and subject to institutional and ethical restrictions. Analysis code supporting the main results is available in Zenodo [23].

## Supplementary Materials

The following supporting information can be downloaded at: https://www.mdpi.com/article/10.3390/genes17060663/s1, Methods S1: Panel derivation, compression, and public-stage adjudication protocol; Methods S2: Single-cell anchoring addendum; Methods S3: External immune-state comparison in GSE224705; Figure S1: Gene-level RT-qPCR support for the locked 6-gene panel in the local PBMC cohort; Figure S2: Full stability assessment of the aggregate-level k = 2 discovery working solution; Figure S3: Transparent compact-panel derivation workflow; Figure S4: Backup-panel sensitivity comparison in the local PBMC cohort; Figure S5: Gene-level RT-qPCR profiles of the locked 6-gene panel in the expanded additional local whole-blood cohort; Table S1: Public cohort summary and study-layer roles; Table S2: Public-stage validation summary and GSE138458 post hoc all-336 sensitivity analysis for adjudication of pre6-any and pre6-balanced; Table S3: Retained endpoint domains and locked cohort-level public-stage adjudication outputs; Table S4: Exact statistical results and analysis classification for local PBMC analyses; Table S5: Treatment-adjusted sensitivity analyses for local PBMC validation; Table S6: Local PBMC cohort comparison of pre6-balanced and pre6-any (backup panel sensitivity analysis); Table S7: Exact statistical and robustness results for the expanded additional local whole-blood validation set; Table S8: Available clinical characteristics of the expanded additional local whole-blood RT-qPCR validation set; Table S9: GSE224705 signature-level and overlap-removed external immune-state comparison of the locked compact score; Table S10: Exploratory response-stratified analysis of the locked compact score in GSE224705; Table S11: Post hoc benchmarking of the locked compact score against a fixed interferon signature and standard clinical markers; Table S12: Post hoc single-gene and reduced-score benchmarking of the locked compact score panel.
