# Supplementary material for: A Staged Whole-Blood Transcriptomic Framework Identifies a Compact Myeloid–Lymphoid Activity Score in Systemic Lupus Erythematosus
Source: Genes (Basel). 2026 Jun 6;17(6):663. doi: 10.3390/genes17060663 (PMC13300284; doi:10.3390/genes17060663)
Supplement: Supplementary file 1 [file genes-17-00663-s001.zip › genes-4341813-supplementary.pdf]

Additional file 1 — Supplementary Material

Table of Contents

Additional Methods

Methods S1. Panel derivation, compression, and public-stage adjudication protocol

Methods S2. Single-cell anchoring addendum

Methods S3. External immune-state comparison in GSE224705

Additional Figures

Figure S1. Gene-level RT-qPCR support for the locked 6-gene panel in the local PBMC cohort

Figure S2. Full stability assessment of the aggregate-level k = 2 discovery working solution

Figure S3. Transparent compact-panel derivation workflow

Figure S4. Backup-panel sensitivity comparison in the local PBMC cohort

Figure S5. Gene-level RT-qPCR profiles of the locked 6-gene panel in the expanded additional local whole-blood cohort

Additional Tables

Table S1. Public cohort summary and study-layer roles

Table S2. Public-stage validation summary and GSE138458 post hoc all-336 sensitivity analysis for adjudication of pre6-any and pre6-balanced

Table S3. Retained endpoint domains and locked cohort-level public-stage adjudication outputs

Table S4. Exact statistical results and analysis classification for local PBMC analyses

Table S5. Treatment-adjusted sensitivity analyses for local PBMC validation

Table S6. Local PBMC cohort comparison of pre6-balanced and pre6-any (backup panel sensitivity analysis)

Table S7. Exact statistical and robustness results for the expanded additional local whole-blood validation set

Table S8. Available clinical characteristics of the expanded additional local whole-blood RT-qPCR validation set

Table S9. GSE224705 signature-level and overlap-removed external immune-state comparison of the locked compact score

Table S10. Exploratory response-stratified analysis of the locked compact score in GSE224705

Table S11. Post hoc benchmarking of the locked compact score against a fixed interferon signature and standard clinical markers

Table S12. Post hoc single-gene and reduced-score benchmarking of the locked compact score panel

Additional Methods

Methods S1. Panel derivation, compression, and public-stage adjudication protocol

Candidate source pool and directional filtering

Panel derivation was constrained to a locked biologically informed source pool rather than unrestricted transcriptome-wide screening. The locked candidate module pool was first expanded to a gene-level source pool comprising 6,233 genes traced back to the whole-blood discovery-stage module system. Discovery-stage cross-cohort directional consistency was evaluated across the three discovery cohorts using the sign

of the cluster-level mean difference after sign-standardised comparison. For each gene, the dominant direction, the number of available cohorts, and directional consistency flags were recorded, and downstream compression prioritised genes with consistent directional support compatible with the locked lymphoid-versus-myeloid/neutrophil-inflammatory bulk interpretation.

Module provenance and redundancy control

Module provenance was retained during compression through locked category labels inherited from the source pool, including lymphoid B/T/NK, myeloid/monocyte, and neutrophil-inflammatory programmes. Redundancy control used pairwise gene-gene correlation within the pooled discovery matrix. Candidate genes were ranked by larger cross-cohort mean absolute difference, larger maximum absolute difference, fewer source modules, and lower mean absolute correlation. A greedy pruning procedure was applied first at $|\rho| \geq 0.90$ and then at $|\rho| \geq 0.85$.

Stepwise compression and final 6-gene candidates

Following source-pool construction and directional filtering, a balanced 12-gene shortlist and a 10-gene shortlist were generated, followed by an 8-gene shortlist for the final 8→6 transition. Leave-one-gene-out analysis identified removable genes. This produced two locked 6-gene candidates: pre6-any (MMP9, MYL9, MME, HAL, CTLA4, CD40LG) and pre6-balanced (MMP9, MYL9, HAL, CTLA4, CD40LG, VPREB3).

Probe handling, sign alignment, and score construction

For probe-based validation cohorts, probe-to-gene annotation and duplicate-gene collapse were performed before score calculation. The expression matrix was organised as genes × samples. Within each cohort, each locked gene was z-standardised across samples using gene-level standardisation: $z_{gi} = (x_{gi} - \mu_g) / \sigma_g$, where x_{gi} denotes the expression value of gene g in sample i, and μ_g and σ_g denote the mean and standard deviation of gene g across samples in that cohort. Gene-level z-scores were then multiplied by predefined direction coefficients so that higher sign-aligned values consistently represented the myeloid/neutrophil-inflammatory side of the axis. For the locked pre6-balanced panel, MMP9, MYL9 and HAL were positively aligned, whereas CTLA4, CD40LG and VPREB3 were negatively aligned. The compact score for each sample was calculated as the unweighted arithmetic mean of the six sign-aligned gene-level z-scores. Standardisation was performed separately within each cohort or validation dataset; therefore, absolute score values were interpreted within each validation layer rather than compared directly across cohorts or blood compartments. For qPCR-based validation, expression-oriented gene-level quantities were used before within-cohort z-standardisation.

Public-stage adjudication rule

Final panel locking was based on public-stage evidence rather than discovery-stage performance alone. GSE138458 served as the primary validation cohort, GSE65391 served as the main extended-validation adjudication cohort, GSE110685 served as a cross-platform RNA-seq support cohort, and GSE61635 was retained as a supportive molecular cohort only because reliable sample-level clinical annotations could not be restored. For GSE65391, the retained endpoint domains comprised the principal SLE-versus-healthy-control comparison together with activity-related or clinical-support endpoints preserved in the finalised one-subject-one-visit analysis. For GSE110685, the retained endpoint domains comprised the principal SLE-versus-healthy-control comparison together with locked vascular phenotype support endpoints. In the finalised manuscript-facing package, retained endpoint-level results were summarised as locked cohort-level adjudication outputs (total_score/rank), and no additional post hoc reweighting was introduced after final locking. Because a standalone endpoint-weight table was not retained, these outputs are reported as locked cohort-level adjudication summaries rather than as a reconstructed endpoint-level scoring formula. Under this framework, both GSE65391 and GSE110685 ranked pre6-balanced above pre6-any, supporting pre6-balanced as the final main panel and retaining pre6-any as a backup/sensitivity panel. As a post hoc sensitivity analysis, the GSE138458 primary-validation workflow was rerun using all 336 architecture-level samples without excluding the six source-designated outliers. This sensitivity analysis did not participate

in panel locking and did not alter the locked final panel definition.

Methods S2. Single-cell anchoring addendum

Single-cell transcriptomic analysis was used exclusively for biological anchoring of the locked bulk-derived final panel and was not used for bulk discovery, public validation model selection, or reverse panel refinement. GSE135779 served as the formal single-cell anchoring dataset. For each annotated cell type, panel-level scores were summarised within SLE and HC groups; the cell-type-specific panel shift was defined as Δscore = mean(panel score in SLE) − mean(panel score in HC). No cell-level inferential testing was applied. For pre6-balanced, the largest positive shifts were observed in monocyte/myeloid ($\Delta = 0.377$), GMP ($\Delta = 0.306$), NK cells ($\Delta = 0.278$), B lineage ($\Delta = 0.256$), HSC ($\Delta = 0.215$), B cells ($\Delta = 0.211$), T cells ($\Delta = 0.203$), and neutrophils ($\Delta = 0.120$). These results were interpreted descriptively as biological anchoring only. GSE174188 is listed as a single-cell reference/resource dataset at the architecture level; the formal main anchoring conclusion was based only on GSE135779.

Methods S3. External immune-state comparison in GSE224705

GSE224705 was used as an external immune-state comparison cohort after the final pre6-balanced panel had been locked. The locked compact score was applied without retraining, threshold optimisation, gene replacement, or model refitting. Predefined IFN, neutrophil/myeloid, and lymphoid T/B-cell signature scores were calculated using prespecified marker sets. Signature-level correlation analyses were performed at both sample and patient levels. Compact-score states and IFN-high/low states were defined using median splits for descriptive overlap analysis, and Fisher’s exact test was used to evaluate overlap between compact-score states and IFN-high/low states. Signature-profile heatmaps were used for descriptive immune-state comparison. Because the reference immune signatures were used for biological contextualisation rather than independent validation, limited overlap between the locked compact panel and the predefined immune-axis marker sets was allowed and was interpreted cautiously at the signature-axis level. As a sensitivity analysis, reference immune signatures were recalculated after removing genes overlapping with the locked compact panel, while the locked compact score itself remained unchanged.

Response-stratified analyses by drug stratum were performed only as exploratory supplementary analyses. These analyses were not used for panel derivation, public-stage adjudication, model refitting, threshold optimisation, treatment-response prediction, or drug-selection inference.

Additional Figures

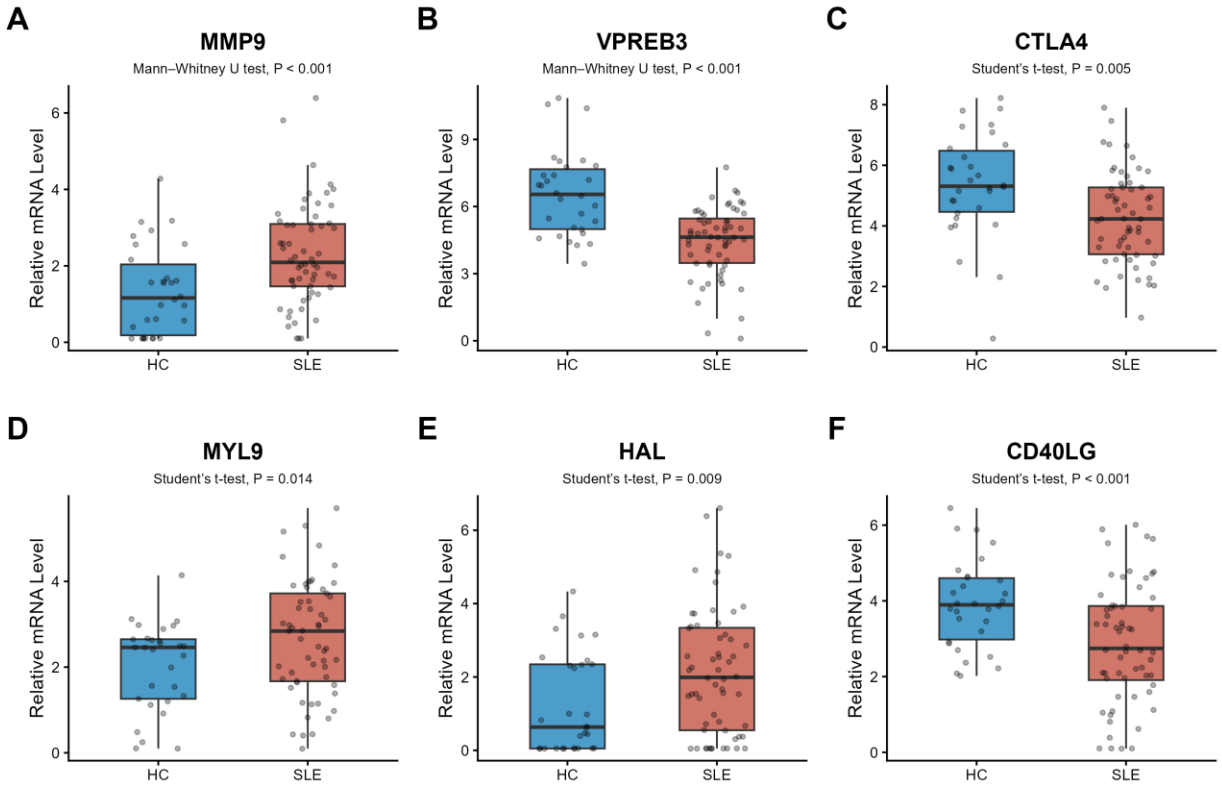

Figure S1. Gene-level RT-qPCR support for the locked 6-gene panel in the local PBMC cohort.

(A–F) RT-qPCR comparisons of the six locked panel genes between SLE patients and HC in the local PBMC cohort. Genes shown: MMP9, VPREB3, CTLA4, MYL9, HAL, and CD40LG. MMP9 ($P < 0.001$) and VPREB3 ($P < 0.001$) were compared using the Mann–Whitney U test; CTLA4 ($P = 0.005$), MYL9 ($P = 0.014$), HAL ($P = 0.009$), and CD40LG ($P < 0.001$) were compared using unpaired two-tailed t-tests. All six genes showed directionally concordant between-group differences, providing supportive evidence for preservation of the locked panel signal in an independent PBMC validation compartment. Main-text interpretation emphasises the compact composite score rather than individual gene-level findings.

Abbreviations: HC, healthy control; PBMC, peripheral blood mononuclear cell; RT-qPCR, reverse transcription quantitative polymerase chain reaction; SLE, systemic lupus erythematosus.

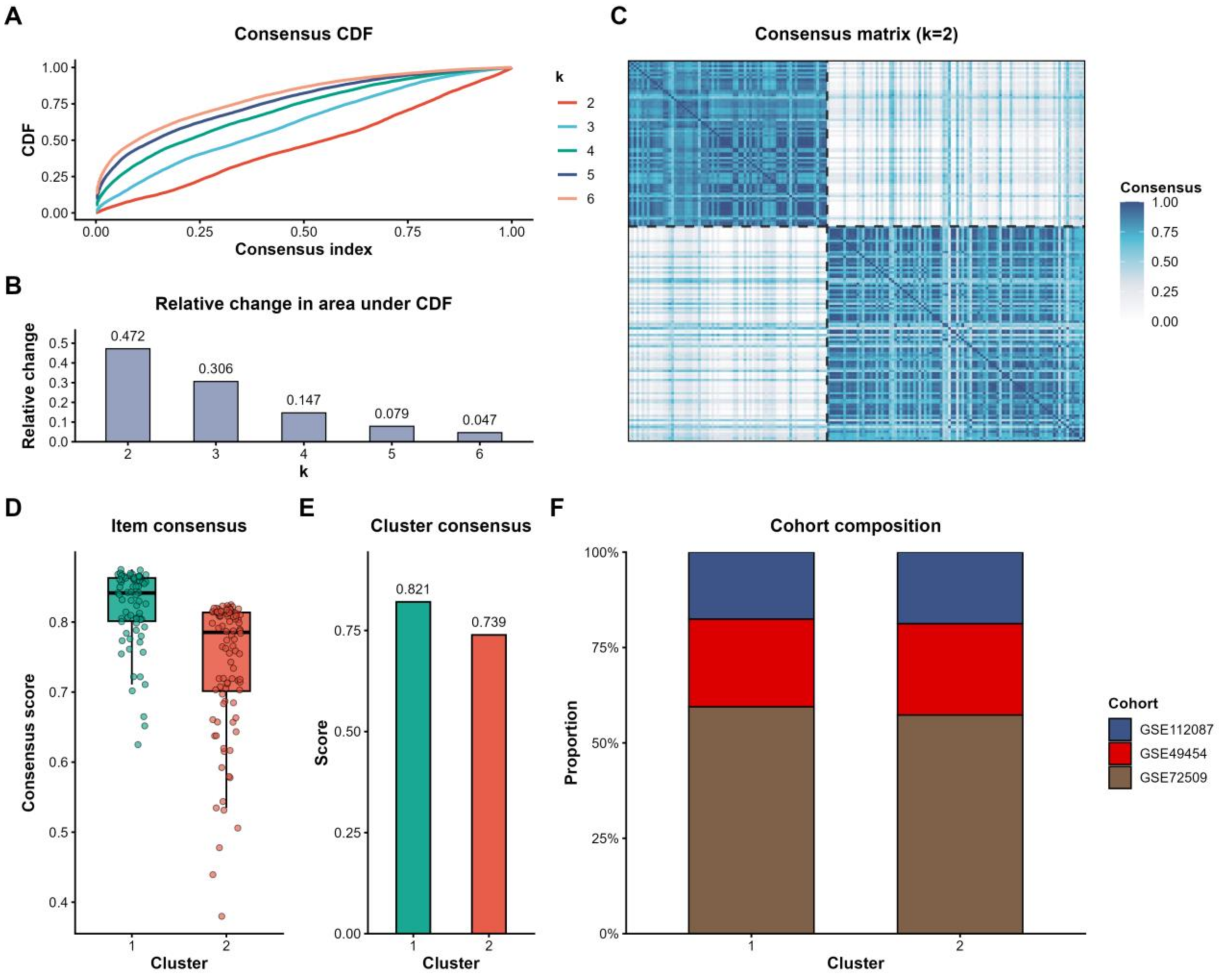

Figure S2. Full stability assessment of the aggregate-level k = 2 discovery working solution.

(A) Consensus CDF curves across candidate k values.

(B) Relative change in area under the consensus CDF. The largest gain occurs at k = 2 (0.472), with diminishing returns at higher k values (k = 3: 0.306; k = 4: 0.147; k = 5: 0.079; k = 6: 0.047).

(C) Consensus matrix for the k = 2 configuration, showing clear block structure.

(D) Item-consensus summary for the k = 2 solution (cluster 1 median ≈ 0.82; cluster 2 median ≈ 0.74).

(E) Cluster-consensus summary (cluster 1: 0.821; cluster 2: 0.739).

(F) Cohort composition of the two k = 2 discovery clusters, showing balanced representation of all three discovery cohorts.

Abbreviations: CDF, cumulative distribution function; SLE, systemic lupus erythematosus. Key stability and cohort-balance panels are summarised in Fig 2C–D.

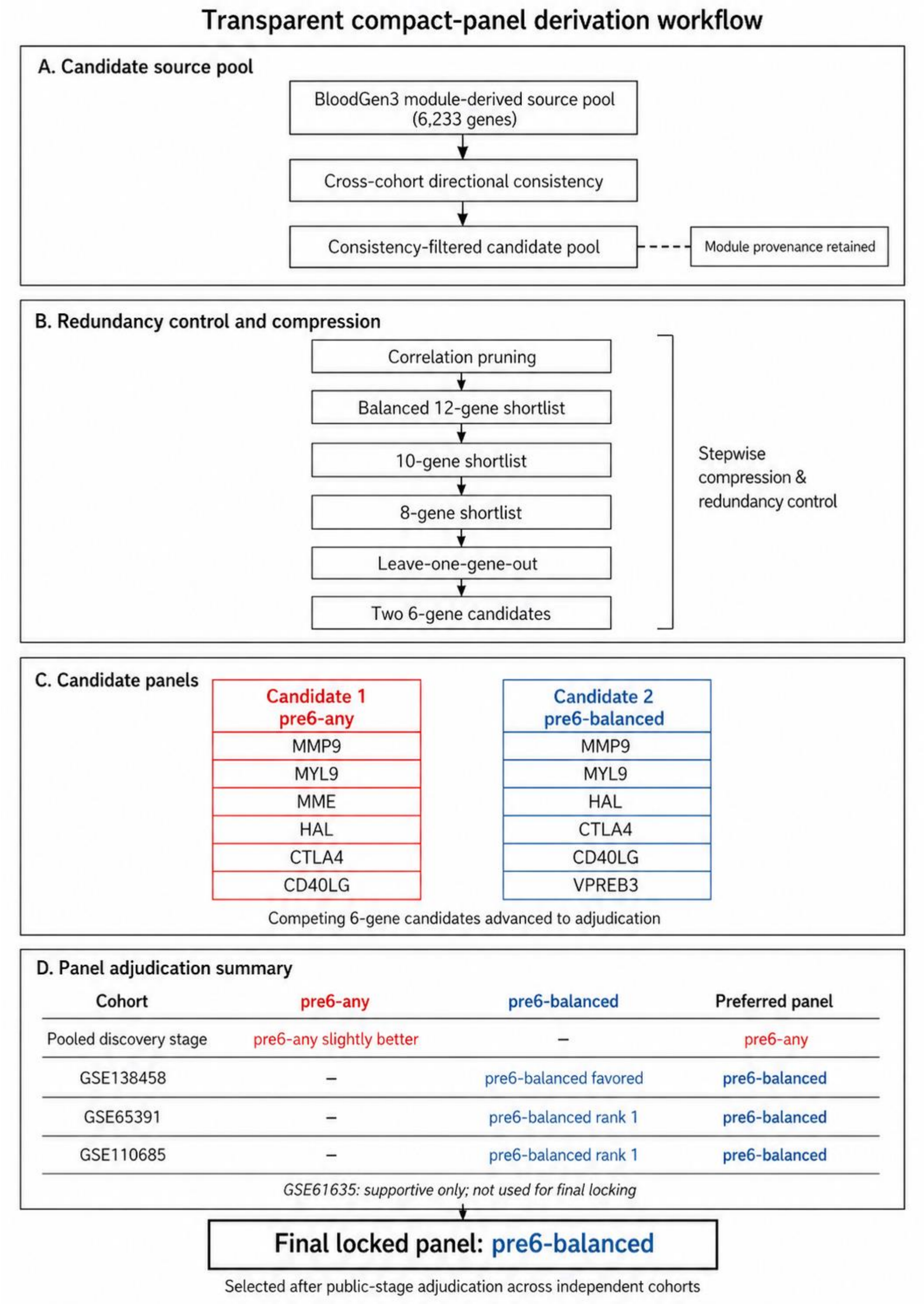

Figure S3. Transparent compact-panel derivation workflow.

A BloodGen3 module-derived source pool of 6,233 genes was filtered by cross-cohort directional consistency with module provenance retained. Redundancy control and stepwise compression yielded two competing 6-gene candidate panels (pre6-any and pre6-balanced). Although pre6-any was marginally favoured at the discovery stage, the integrated public-stage adjudication favoured pre6-balanced,

particularly across the major extended-validation cohorts, and pre6-balanced was locked as the final panel. A post hoc all-336 sensitivity analysis in GSE138458 is reported in Table S2. GSE61635 was retained as a supportive molecular cohort only.

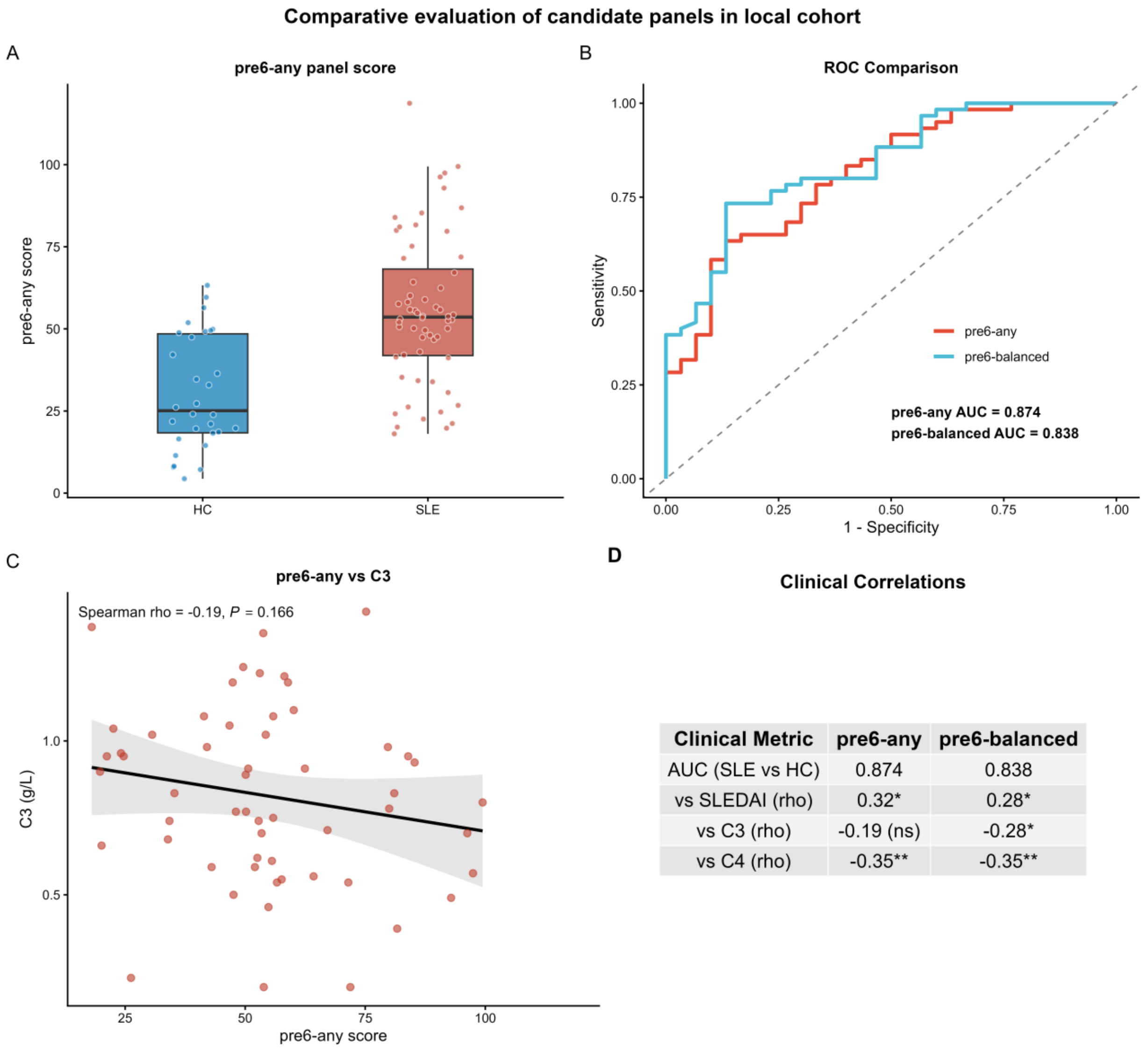

Figure S4. Backup-panel sensitivity comparison in the local PBMC cohort.

(A) Comparison of the pre6-any backup panel score between HC and SLE in the local PBMC cohort.

(B) ROC comparison of pre6-any and the final locked pre6-balanced panel for SLE-versus-HC discrimination.

(C) Correlation between the pre6-any backup panel score and C3. (D) Summary of selected clinical associations comparing pre6-any with the final locked pre6-balanced panel. These analyses were performed only as supplementary backup-panel sensitivity analyses and were not used for final panel selection. The final main panel remained pre6-balanced based on the locked public-stage adjudication framework.

Abbreviations: AUC, area under the receiver operating characteristic curve; C3, complement component 3; HC, healthy control; PBMC, peripheral blood mononuclear cell; ROC, receiver operating characteristic; SLE, systemic lupus erythematosus.

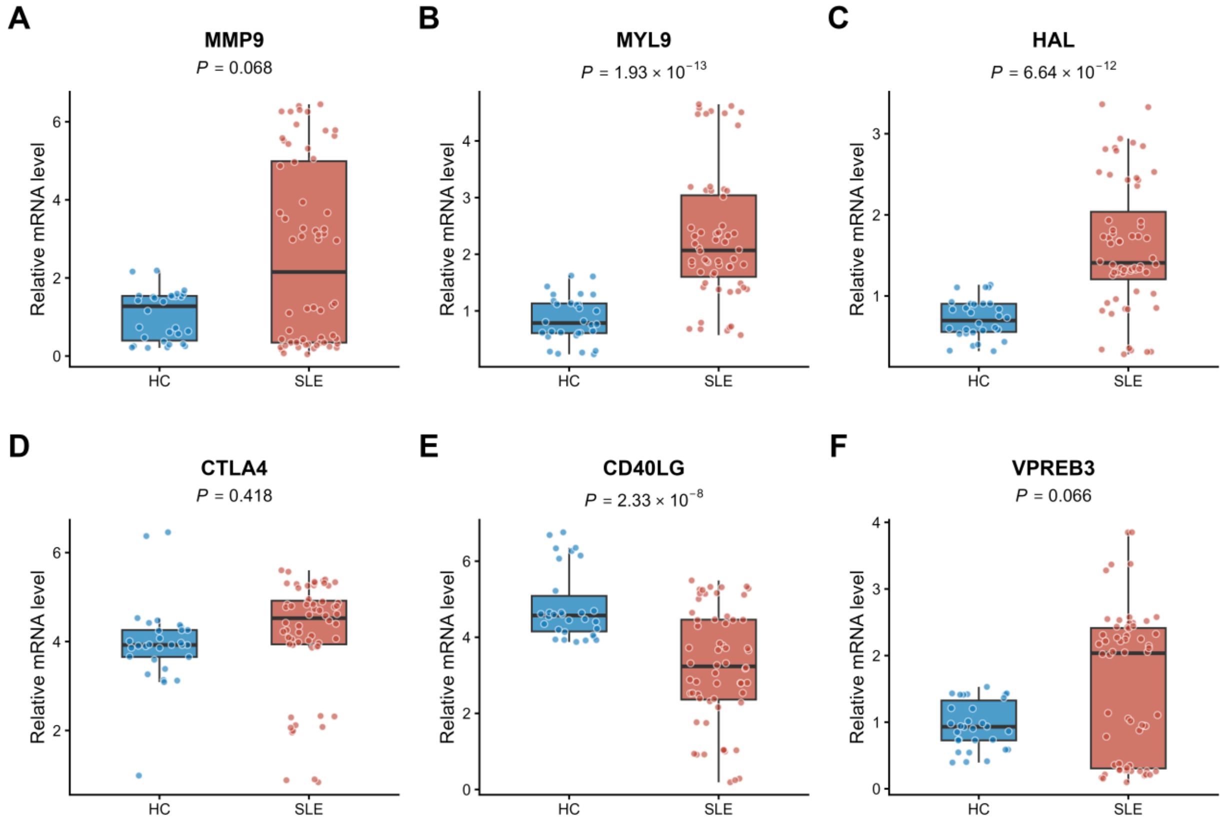

Figure S5. Gene-level RT-qPCR profiles of the locked 6-gene panel in the expanded additional local whole-blood cohort.

Gene-level RT-qPCR profiles of the six locked pre6-balanced panel genes (MMP9, MYL9, HAL, CTLA4, CD40LG, VPREB3) were evaluated in the expanded additional local whole-blood RT-qPCR validation set (30 HC and 60 SLE). MYL9 ($P = 1.93 \times 10^{-13}$), HAL ($P = 6.64 \times 10^{-12}$), and CD40LG ($P = 2.33 \times 10^{-8}$) showed statistically significant between-group differences, whereas MMP9 ($P = 0.068$) and VPREB3 ($P = 0.066$) showed borderline trends and CTLA4 ($P = 0.418$) did not differ significantly. These gene-level results were interpreted as exploratory expression profiles rather than as independent criteria for panel refinement. The primary whole-blood validation was based on the locked composite compact score shown in Fig 5C–F; a condensed gene-level summary is shown in Fig 5G.

Abbreviations: HC, healthy control; RT-qPCR, reverse transcription quantitative polymerase chain reaction; SLE, systemic lupus erythematosus.

Additional Tables

Table S1. Public cohort summary and study-layer roles.

Stage	Dataset	Platform	Total n	SLE n	HC n	Sample type	Main role
Discovery	GSE72509	RNA-seq	117	99	18	Whole blood	Core discovery
Discovery	GSE112087	RNA-seq	60	31	29	Whole blood	Core discovery
Discovery	GSE49454	Microarray	78*	58	20	Whole blood	Discovery (annotated)

Stage	Dataset	Platform	Total n	SLE n	HC n	Sample type	Main role
Primary validation	GSE138458	Microarray	336	312	24	Whole blood	Primary validation
Extended	GSE65391	Microarray	996	924	72	Whole blood	Extended validation
Extended	GSE110685	RNA-seq	53	36	17	Whole blood	Extended validation
Extended	GSE61635	Microarray	129	99	30	Whole blood	Supportive only
Single-cell	GSE135779	scRNA-seq	58	41	17	PBMC	Formal SC anchoring
Single-cell	GSE174188	scRNA-seq	261	162	99	PBMC	SC reference/resource
Reserve	GSE20864	Microarray	66	21	45	Peripheral blood	Reserve only

*GSE49454 architecture-level display: 78 samples. Downstream discovery analyses used the stricter locked analytic subset (56 samples; total discovery analysis set: 233 samples). Single-cell datasets are reference/resource datasets for biological anchoring only. GSE138458 is shown at the architecture level as 336 samples (312 SLE and 24 HC); the locked primary-validation compact-score analysis used an outlier-removed subset of 330 samples (307 SLE and 23 HC).

Table S2. Public-stage validation summary and GSE138458 post hoc all-336 sensitivity analysis for adjudication of pre6-any and pre6-balanced.

Part A. Public-stage validation summary for adjudication of pre6-any and pre6-balanced.

Cohort	Validation tier	Analysis contrast	Sample design/note	pre6-any	pre6-balanced	Preferred panel	Used for final adjudication
GSE138458	Primary	SLE vs HC; High vs Low activity	Outlier-removed subset: 23 HC, 307 SLE; activity endpoint: 156 high, 151 low	Case vs HC: AUC = 0.659, d = 0.616	Case vs HC: AUC = 0.712, d = 0.731	pre6-balanced	Yes
GSE65391	Extended	SLE vs HC; activity endpoints	One-subject-one-visit design	Rank 2, total_score 1.68	Rank 1, total_score 1.97	pre6-balanced	Yes
GSE110685	Extended	SLE vs HC; vascular phenotype	Whole-blood RNA-seq; 36 SLE, 17 HC	Rank 2, total_score 1.00	Rank 1, total_score 3.00	pre6-balanced	Yes
GSE61635	Supportive	SLE vs HC	Clinical annotations not recoverable	Significant	Significant	Supportive only	No
Overall	Integrated	All tiers	Discovery slightly favoured pre6-any; public-stage evidence prioritised	Backup/sensitivity panel	Final main panel	pre6-balanced	Yes

Note: This table summarises public-stage evidence used to adjudicate the two locked 6-gene candidate panels. “Used for final adjudication” indicates whether the cohort contributed to the locked public-stage final panel decision. GSE61635 was retained as supportive molecular evidence only and was not used for principal final locking because reliable sample-level clinical annotations could not be restored. The final main panel remained pre6-balanced; pre6-any was retained as a backup/sensitivity panel. For GSE138458, Table S1 reports architecture-level cohort allocation, whereas Table S2 reports the outlier-removed primary-validation analysis subset used for compact-score comparison. The six GSE138458 samples excluded from the predefined primary-validation compact-score analysis were GSM4109031 (200319680117_E; SLE), GSM4109192 (200308380073_J; SLE), GSM4109193 (200308380073_K; SLE), GSM4109194 (200308380073_L; HC), GSM4109223 (200308380104_E; SLE), and GSM4109271 (200667730029_E; SLE). These samples were listed as source-designated outliers and were dropped prior

to normalisation. A separate finalised numeric QC-threshold table was not retained in the manuscript-facing package. In Part A, the GSE138458 “Used for final adjudication” entry refers only to the predefined outlier-removed primary-validation analysis, not to the post hoc all-336 sensitivity analysis reported in Parts C–D.

Abbreviations: AUC, area under the receiver operating characteristic curve; HC, healthy control; RNA-seq, RNA sequencing; SLE, systemic lupus erythematosus.

Part B. Excluded GSE138458 samples and source-designated QC reason.

GSM ID	Sample ID	Subject ID	SLE/HC	Activity label	Excluded from main analysis	Locked QC reason
GSM4109031	200319680117_E	425	SLE	High	Yes	Source-designated outlier
GSM4109192	200308380073_J	31	SLE	High	Yes	Source-designated outlier
GSM4109193	200308380073_K	177	SLE	Low	Yes	Source-designated outlier
GSM4109194	200308380073_L	541728	HC	Not applicable	Yes	Source-designated outlier
GSM4109223	200308380104_E	360	SLE	Low	Yes	Source-designated outlier
GSM4109271	200667730029_E	472	SLE	High	Yes	Source-designated outlier

The six excluded samples were identified in the source processing record as outliers and were dropped prior to normalisation. Of these, five were SLE samples (three high-activity and two low-activity) and one was a healthy control without an activity label.

Part C. GSE138458 post hoc all-336 sensitivity analysis.

Endpoint	Panel	n	Group 1	Group 2	Gr1 mean ± SD	Gr2 mean ± SD	Gr1 median (IQR)	Gr2 median (IQR)	AUC (95% CI)	Cohen's d	Wilcoxon P
SLE vs HC	pre6-any	336	HC (n=24)	SLE (n=312)	−0.420 ± 0.534	0.032 ± 0.723	−0.556 (0.817)	0.193 (1.254)	0.684 (0.600–0.769)	0.636	0.00264
SLE vs HC	pre6-balanced	336	HC (n=24)	SLE (n=312)	−0.298 ± 0.537	0.023 ± 0.809	−0.360 (0.523)	0.198 (1.447)	0.627 (0.547–0.706)	0.405	0.0387
High vs Low activity	pre6-any	312	Low (n=153)	High (n=159)	0.018 ± 0.704	0.046 ± 0.743	0.150 (1.219)	0.259 (1.298)	0.512 (0.447–0.576)	0.038	0.720
High vs Low activity	pre6-balanced	312	Low (n=153)	High (n=159)	−0.002 ± 0.787	0.047 ± 0.831	0.132 (1.280)	0.292 (1.520)	0.527 (0.462–0.591)	0.061	0.417

AUC 95% confidence intervals were calculated using the DeLong method. The formal primary-validation results were based on the predefined outlier-removed subset (n = 330), whereas the all-336 analysis was performed post hoc as a sensitivity analysis only and was not used for panel locking.

Part D. Comparison of the locked n=330 and post hoc all-336 analyses in GSE138458.

Endpoint	Analysis set	pre6-any	pre6-balanced	Directional conclusion
SLE vs HC	Locked primary analysis (n=330)	AUC = 0.6591; Cohen's d = 0.6161; P = 0.0109	AUC = 0.7118; Cohen's d = 0.7306; P = 0.000705	pre6-balanced favoured
SLE vs HC	All-sample sensitivity (n=336)	AUC = 0.6842 (95% CI 0.5998–0.7687); Cohen's d = 0.6363; P = 0.00264	AUC = 0.6267 (95% CI 0.5472–0.7061); Cohen's d = 0.4051; P = 0.0387	pre6-any favoured
High vs Low activity	Locked primary analysis (n=307 SLE; High 156 / Low 151)	AUC = 0.5128; Cohen's d = 0.0530; P = 0.6982	AUC = 0.5170; Cohen's d = 0.0733; P = 0.6065	pre6-balanced slightly favoured; both weak
High vs Low activity	All-sample sensitivity (n=312 SLE; High 159 / Low 153)	AUC = 0.5118 (95% CI 0.4474–0.5761); Cohen's d = 0.0383; P = 0.7196	AUC = 0.5266 (95% CI 0.4622–0.5910); Cohen's d = 0.0612; P = 0.4174	pre6-balanced slightly favoured; both weak

The all-336 analysis was performed post hoc as a sensitivity analysis and was not used for panel locking. The locked primary-validation analysis used the predefined outlier-removed subset (n = 330). In the all-336 sensitivity analysis of GSE138458, the relative advantage of pre6-balanced was not maintained for the

case-control endpoint, whereas the activity-related comparison remained weak and directionally similar.

Table S3. Retained endpoint domains and locked cohort-level public-stage adjudication outputs.

Cohort	Retained endpoint domains	Endpoint-level interpretation retained in locked package	Locked cohort-level output	Final use in adjudication
GSE138458	SLE vs HC; high vs low activity	In the predefined outlier-removed primary-validation subset, the case-vs-control comparison favoured pre6-balanced over pre6-any; the activity-related comparison was weak for both panels but directionally favoured pre6-balanced. A post hoc all-336 sensitivity analysis did not maintain the case-control preference for pre6-balanced.	Direct endpoint metrics; not summarised as total_score/rank	Yes
GSE65391	SLE vs HC; activity-related / clinical-support endpoints	Retained endpoint domains supported pre6-balanced at cohort level	pre6-balanced: total_score = 1.97, rank = 1; pre6-any: total_score = 1.68, rank = 2	Yes
GSE110685	SLE vs HC; vascular phenotype support endpoints	Retained endpoint domains supported pre6-balanced at cohort level	pre6-balanced: total_score = 3.00, rank = 1; pre6-any: total_score = 1.00, rank = 2	Yes
GSE61635	SLE vs HC only	Both panels showed molecular SLE-vs-HC support; clinical-layer adjudication unavailable	Supportive only	No
Overall decision	Primary + major extended-validation evidence	Discovery slightly favoured pre6-any, but public-stage evidence converged on pre6-balanced	pre6-balanced retained as final main panel; pre6-any retained as backup/sensitivity panel	Yes

For GSE65391 and GSE110685, retained endpoint-level results were summarised as locked cohort-level total_score/rank outputs. Because a standalone endpoint-weight table was not retained in the manuscript-facing package, this table reports retained endpoint domains and locked cohort-level adjudication outputs rather than a reconstructed endpoint-level scoring formula. Local PBMC and whole-blood qPCR validation results were not used for final panel locking.

Abbreviations: HC, healthy control; RNA-seq, RNA sequencing; SLE, systemic lupus erythematosus.

Table S4. Exact statistical results and analysis classification for local PBMC analyses.

Figure panel	Variable / comparison	Statistical method	Effect size	P value	FDR q value	Testing basis	Analysis type
Fig 4A	Score (SLE vs HC)	Unpaired t-test	d = 1.62	1.4×10^{-8}	5.95×10^{-8}	Passed Shapiro–Wilk & Levene's	Prespecified supportive
Fig 4C	Score (High vs Low/mod)	Mann–Whitney U	r = 0.33	0.0055	0.0079	Non-parametric	Prespecified supportive

Figure panel	Variable / comparison	Statistical method	Effect size	P value	FDR q value	Testing basis	Analysis type
Fig 4E	Score vs SLEDAI	Spearman correlation	ρ = 0.28	0.029	0.0329	Non-parametric	Prespecified supportive
Fig 4F	Score vs C3	Spearman correlation	ρ = −0.28	0.039	0.0414	Non-parametric	Exploratory
Fig 4G	Score vs C4	Spearman correlation	ρ = −0.35	0.0056	0.0079	Non-parametric	Exploratory
Fig 5A	Score vs Classical monocytes (%)	Spearman correlation	ρ = −0.51	3.2×10^{-5}	9.07×10^{-5}	Non-parametric	Exploratory
Fig 5A	Score vs Intermediate monocytes (%)	Spearman correlation	ρ = 0.41	0.0024	0.0045	Non-parametric	Exploratory
Fig 5A	Score vs Nonclassical monocytes (%)	Spearman correlation	ρ = 0.41	0.0013	0.0028	Non-parametric	Exploratory
Fig 5A	Score vs $CD169^+$ monocytes (%)	Spearman correlation	ρ = 0.60	4.1×10^{-7}	1.39×10^{-6}	Non-parametric	Exploratory
Fig S1A	MMP9 mRNA level	Mann–Whitney U	r = 0.45	<0.001	5.67×10^{-12}	Failed normality	Exploratory
Fig S1B	VPREB3 mRNA level	Mann–Whitney U	r = 0.48	<0.001	5.67×10^{-12}	Failed normality	Exploratory
Fig S1C	CTLA4 mRNA level	Unpaired t-test	d = 0.52	0.005	0.0079	Passed normality	Exploratory
Fig S1D	MYL9 mRNA level	Unpaired t-test	d = 0.38	0.014	0.017	Passed normality	Exploratory
Fig S1E	HAL mRNA level	Unpaired t-test	d = 0.41	0.009	0.0118	Passed normality	Exploratory
Fig S1F	CD40LG mRNA level	Unpaired t-test	d = 0.58	<0.001	2.43×10^{-4}	Passed normality	Exploratory
Fig S4C	pre6-any vs C3	Spearman correlation	ρ = −0.21	0.122	0.122	Non-parametric (ns)	Backup-panel sensitivity

Abbreviations: AUC, area under the receiver operating characteristic curve; FDR, false discovery rate; HC, healthy control; PBMC, peripheral blood mononuclear cell; SLE, systemic lupus erythematosus; SLEDAI, Systemic Lupus Erythematosus Disease Activity Index.

Table S5. Treatment-adjusted sensitivity analyses for local PBMC validation.

Association	Adjusted variables	Statistical method	Adjusted effect size	P value
Compact score vs SLEDAI	Prednisone-equivalent dose + major immunosuppressant use	Partial Spearman correlation	ρ = 0.25	0.041
Compact score vs C3	Prednisone-equivalent dose + major immunosuppressant use	Partial Spearman correlation	ρ = −0.24	0.048
Compact score vs C4	Prednisone-equivalent dose + major immunosuppressant use	Partial Spearman correlation	ρ = −0.31	0.009

Note: Partial Spearman correlations were adjusted for prednisone-equivalent dose and major immunosuppressant use. These analyses were performed as treatment-adjusted sensitivity analyses for the locked compact score in the local PBMC validation cohort.

Abbreviations: C3, complement component 3; C4, complement component 4; SLEDAI, Systemic Lupus Erythematosus Disease Activity Index.

Table S6. Local PBMC cohort comparison of pre6-balanced (final locked panel) and pre6-any (backup panel).

Endpoint	pre6-balanced (locked)	pre6-any (backup)	Comment
SLE vs HC: AUC (95% CI)	0.838 [0.754, 0.922]	0.874 [0.796, 0.938]	pre6-any numerically higher
SLE vs HC: Cohen's d	1.62	1.54	Both large
High vs Low activity (P)	0.0055	0.0119	Both significant; pre6-balanced stronger
Score vs SLEDAI (ρ, P)	ρ = 0.28, P = 0.029	ρ = 0.32, P = 0.017	Both significant
Score vs C3 (ρ, P)	ρ = −0.28, P = 0.039	ρ = −0.19, P = 0.166 (ns)	pre6-any C3 association non-significant
Score vs C4 (ρ, P)	ρ = −0.35, P = 0.0056	ρ = −0.35, P = 0.007	Comparable

These findings provide supplementary biological context for the backup panel but were not used for panel selection. The final main panel remained pre6-balanced based on the locked public-stage adjudication framework.

Abbreviations: AUC, area under the receiver operating characteristic curve; C3, complement component 3; C4, complement component 4; CI, confidence interval; HC, healthy control; PBMC, peripheral blood mononuclear cell; SLE, systemic lupus erythematosus; SLEDAI, Systemic Lupus Erythematosus Disease Activity Index.

Table S7. Exact statistical and robustness results for the expanded additional local whole-blood validation set.

Analysis	Sample	Method	Result	Interpretation
Score: SLE vs HC	30 HC, 60 SLE	Mann–Whitney U	$P = 2.37 \times 10^{-9}$	Prespecified supportive
ROC: SLE vs HC	30 HC, 60 SLE	ROC analysis	AUC = 0.888, 95% CI 0.821–0.954	Supportive discrimination
Score: High vs Low/mod activity	SLE only (n = 60)	Mann–Whitney U	$P = 3.59 \times 10^{-7}$; low/moderate n = 18, high n = 42	Activity-related support
ROC: High vs Low/mod activity	SLE only (n = 60)	ROC analysis	AUC = 0.918; low/moderate n = 18, high n = 42	Supportive activity discrimination
Score vs SLEDAI	SLE only (n = 60)	Spearman correlation	$\rho = 0.819$, $P = 1.25 \times 10^{-15}$	Strong activity association
Treatment-adjusted score vs SLEDAI	SLE only (n = 60)	Partial Spearman correlation	partial $\rho = 0.814$, $P = 2.58 \times 10^{-15}$	Treatment-adjusted support
Score vs C3	SLE only (n = 60)	Spearman correlation	ρ = −0.058, P = 0.657	Not significant
Score vs C4	SLE only (n = 60)	Spearman correlation	ρ = 0.241, P = 0.063	Exploratory trend (not significant)
Score vs anti-dsDNA	SLE only (n = 60)	Spearman correlation	ρ = 0.196, P = 0.134	Not significant
MMP9 expression	30 HC, 60 SLE	Gene-level test	P = 0.068 (borderline trend)	Exploratory gene-level profile
MYL9 expression	30 HC, 60 SLE	Gene-level test	$P = 1.93 \times 10^{-13}$	Exploratory gene-level profile
HAL expression	30 HC, 60 SLE	Gene-level test	$P = 6.64 \times 10^{-12}$	Exploratory gene-level profile
CTLA4 expression	30 HC, 60 SLE	Gene-level test	P = 0.418 (not significant)	Exploratory gene-level profile
CD40LG expression	30 HC, 60	Gene-level test	$P = 2.33 \times 10^{-8}$	Exploratory gene-level

Analysis	Sample	Method	Result	Interpretation
	SLE			profile
VPREB3 expression	30 HC, 60 SLE	Gene-level test	P = 0.066 (borderline trend)	Exploratory gene-level profile

This expanded whole-blood set was used exclusively for compartment-matched orthogonal supportive validation and was not used for panel derivation, model refitting, or threshold optimisation. Disease activity subgroups: low/moderate n = 18, high n = 42 (SLEDAI > 6 threshold). Treatment-adjusted analysis adjusted for prednisone-equivalent dose, hydroxychloroquine use, and major immunosuppressant use.

Abbreviations: AUC, area under the receiver operating characteristic curve; CI, confidence interval; C3, complement component 3; C4, complement component 4; HC, healthy control; SLE, systemic lupus erythematosus; SLEDAI, Systemic Lupus Erythematosus Disease Activity Index. Gene-level tests correspond to the exploratory gene-level profiles summarised in Fig 5G and shown in full in Fig S5.

Part B. Robustness analyses of the locked whole-blood compact score.

**Analysis**	**Metric**	**Original estimate**	**Sensitivity estimate**	**Interpretation**
Leave-one-out sample sensitivity	Score vs SLEDAI Spearman ρ	0.819	0.811–0.842	Association with SLEDAI was not driven by any single sample.
Bootstrap resampling	SLE vs HC AUC	0.888	95% CI 0.813–0.951	SLE-versus-HC discrimination remained stable across resampling.
Bootstrap resampling	High vs low/moderate activity AUC	0.918	95% CI 0.831–0.980	Activity-related discrimination remained stable across resampling.
Bootstrap resampling	Score vs SLEDAI Spearman ρ	0.819	95% CI 0.704–0.890	Association with disease activity remained stable across resampling.
LOGO	Omit MMP9	—	AUC SLE/HC 0.872; AUC activity 0.817; ρ 0.525	Performance was attenuated but not abolished.
LOGO	Omit MYL9	—	AUC SLE/HC 0.817; AUC activity 0.853; ρ 0.475	Performance was attenuated but not abolished.
LOGO	Omit HAL	—	AUC SLE/HC 0.813; AUC activity 0.844; ρ 0.522	Performance was attenuated but not abolished.
LOGO	Omit CTLA4	—	AUC SLE/HC 0.913; AUC activity 0.849; ρ 0.544	Performance was attenuated but not abolished.
LOGO	Omit CD40LG	—	AUC SLE/HC 0.878; AUC activity 0.866; ρ 0.493	Performance was attenuated but not abolished.
LOGO	Omit VPREB3	—	AUC SLE/HC 0.883; AUC activity 0.919; ρ 0.795	Performance was largely preserved after omission of VPREB3.

Bootstrap confidence intervals were computed using 1,000 resampling iterations. High disease activity was defined as SLEDAI > 6. In the LOGO analysis, the score was recalculated by applying the locked sign-aligned z-score averaging rule to the remaining five genes, without model refitting, gene replacement, or post hoc weighting.

Table S8. Available clinical characteristics of the expanded additional local whole-blood RT-qPCR

validation set.

Characteristic	HC (n = 30)	SLE (n = 60)	P value
Female sex, n (%)	27 (90.0%)	54 (90.0%)	1.000
Age, years	33.00 [30.00–37.00]	34.00 [28.75–40.00]	0.804
SLEDAI	—	9.00 [5.00–13.25]	—
High disease activity (SLEDAI > 6), n (%)	—	42 (70.0%)	—
Low/moderate disease activity, n (%)	—	18 (30.0%)	—
C3, g/L	1.11 [1.04–1.21]	0.81 [0.69–0.96]	3.42×10^{-9}
C4, g/L	0.28 [0.24–0.32]	0.12 [0.10–0.14]	2.84×10^{-14}
anti-dsDNA, IU/mL	18.20 [13.10–20.90]	387.75 [183.57–548.20]	2.59×10^{-13}
Prednisone-equivalent dose, mg/day	0.00 [0.00–0.00]	10.00 [5.00–15.00]	1.05×10^{-12}
Hydroxychloroquine use, n (%)	0 (0.0%)	52 (86.7%)	7.27×10^{-17}
Immunosuppressant use, n (%)	0 (0.0%)	36 (60.0%)	2.21×10^{-9}
Locked whole-blood compact score	−0.495 [−0.572 to −0.399]	0.217 [−0.143 to 0.806]	2.37×10^{-9}

Data presented as median [IQR] for continuous variables and n (%) for categorical variables. High disease activity defined as SLEDAI > 6. This expanded whole-blood set (30 HC, 60 SLE) was independent of the local PBMC validation cohort with no overlapping participants, and was used only for compartment-matched supportive validation.

Abbreviations: C3, complement component 3; C4, complement component 4; HC, healthy control; IQR, interquartile range; SLE, systemic lupus erythematosus; SLEDAI, Systemic Lupus Erythematosus Disease Activity Index.

Table S9. GSE224705 signature-level and overlap-removed external immune-state comparison of the locked compact score.

The locked pre6-balanced compact score was applied without retraining, threshold optimisation, or gene-panel modification. Signature scores were calculated using predefined marker sets; state labels used median splits for descriptive comparison. These reference signatures were used for immune-axis contextualisation rather than independent validation; limited gene overlap with the locked compact panel was therefore interpreted cautiously at the signature-axis level.

Part A. Locked genes and predefined signature marker sets.

Category	Predefined genes	Available/used genes	Availability	Analysis role	Note
Locked compact score	MMP9, MYL9, HAL, CTLA4, CD40LG, VPREB3	6/6	All included	Final locked pre6-balanced panel	Higher score represents the myeloid/neutrophil-inflammatory side after sign alignment.
IFN signature	IFI27, IFI44L, IFIT1, ISG15, MX1, OAS1	6/6	All used	Canonical IFN comparison	Used to test whether the compact score duplicates IFN-high/low classification.
Neutrophil/myeloid signature	MMP9, S100A8, S100A9, FCGR3B, CEACAM8	5/5	All used	Myeloid reference signature	Used to assess alignment with neutrophil/myeloid inflammatory

Category	Predefined genes	Available/used genes	Availability	Analysis role	Note
					activity.
Lymphoid T/B-cell signature	CD3D, CD3E, CD79A, MS4A1, CD40LG, CTLA4	6/6	All used	Lymphoid reference signature	Used to assess inverse alignment with lymphoid T/B-cell activity.

Part B. Signature correlation analysis.

Comparison	Sample-level ρ	Sample-level P	Patient-level ρ	Patient-level P	Interpretation
Compact score vs IFN signature	0.102	0.0349	0.0185	0.814	Weak/minimal association; not an IFN-only surrogate.
Compact score vs neutrophil/myeloid signature	0.733	$<2\times10^{-16}$	0.760	$<2\times10^{-16}$	Strong positive association with neutrophil/myeloid activity.
Compact score vs lymphoid T/B-cell signature	−0.834	$<2\times10^{-16}$	−0.833	$<2\times10^{-16}$	Strong inverse association with lymphoid T/B-cell activity.

Part C. Compact-state vs IFN-high/low overlap.

Compact-score state	IFN-high n (%)	IFN-low n (%)	Fisher exact P	Interpretation
Compact-low (lymphoid-side)	106 (49.5%)	108 (50.5%)	0.923	Near-even IFN-high/low distribution.
Compact-high (myeloid-side)	108 (50.5%)	106 (49.5%)	0.923	Near-even IFN-high/low distribution.

The Fisher exact P value applies to the overall 2 × 2 overlap between compact-score states and IFN-high/low states.

Abbreviations: IFN, interferon; SLE, systemic lupus erythematosus.

Part D. Overlap-removed signature sensitivity analysis.

Reference signature	Removed overlapping genes	Retained genes	Sample-level ρ	Sample-level P	Patient-level ρ	Patient-level P	Interpretation
IFN	None	IFI27, IFI44L, IFIT1, ISG15, MX1, OAS1	0.102	0.0348	0.0185	0.814	Weak/minimal association.
Neutrophil/myeloid	MMP9	S100A8, S100A9, FCGR3B, CEACAM8	0.640	1.08×10^{-50}	0.673	7.41×10^{-23}	Positive association remained directionally consistent.
Lymphoid T/B-cell	CD40LG, CTLA4	CD3D, CD3E, CD79A, MS4A1	−0.735	5.61×10^{-74}	−0.753	4.48×10^{-31}	Inverse association remained directionally consistent.

In this sensitivity analysis, the locked compact score remained unchanged. Only the reference immune signatures were recalculated after removing genes overlapping with the locked compact panel.

Table S10. Exploratory response-stratified analysis of the locked compact score in GSE224705.

Baseline SRI-4 responder versus non-responder analyses were performed by drug stratum using the locked

compact score. These analyses are exploratory and were not used for panel selection, threshold optimisation, treatment-response prediction, or drug-selection inference.

Drug group	Responder n	Non-responder n	Median score: responder	Median score: non-responder	Wilcoxon P	Cliff's delta	AUC	AUC 95% CI	Interpretation
AZA	11	9	0.052	0.138	0.254	−0.313	0.343	0.087–0.600	No robust separation
HCQ	56	14	−0.206	−0.167	0.382	−0.153	0.423	0.258–0.589	No robust separation
MMF	34	10	0.042	−0.143	0.566	0.124	0.562	0.388–0.735	No robust separation
PHC	17	12	0.133	0.184	0.550	−0.137	0.431	0.203–0.660	No robust separation
SOC	73	26	−0.171	−0.035	0.109	−0.213	0.394	0.264–0.524	No robust separation

HCQ was used to avoid confusion between hydroxychloroquine and healthy controls. SOC is a derived standard-of-care grouping from the parsed metadata. AUC values are descriptive and were not used for treatment-response prediction or drug-selection claims. Abbreviations: AUC, area under the receiver operating characteristic curve; AZA, azathioprine; CI, confidence interval; HCQ, hydroxychloroquine; MMF, mycophenolate mofetil; PHC, pulsed/high-dose corticosteroid; SOC, standard of care.

Table S11. Post hoc benchmarking of the locked compact score against a fixed interferon signature and standard clinical markers.

All analyses are post hoc benchmarking only. They did not alter the locked final panel (pre6-balanced), the score construction formula, or the public-stage adjudication framework. The IFN signature comprised: IFI27, IFI44L, IFIT1, ISG15, MX1, OAS1. Local PBMC benchmarking used the locked high versus low/moderate activity comparison (SLEDAI > 6; n = 60 SLE). GSE224705 benchmarking: SLE/LN n = 428, HC n = 20; high activity SLEDAI ≥ 10, n = 19; low/moderate n = 409. Results do not establish clinical utility or superiority over standard markers.

Section	Readout	Cohort	SLEDAI ρ	SLEDAI P	High vs low/mod AUC (95% CI)	SLE/LN vs HC AUC	Note
Local clinical marker benchmark	Compact score (locked)	Local PBMC	0.281	0.029	0.719 (0.571–0.844)	0.838 (0.754–0.922)	Locked final score; post hoc benchmark only
Local clinical marker benchmark	C3 (inverse direction)	Local PBMC	−0.218	0.106	0.754 (0.610–0.883)	—	Direction-aligned AUC; post hoc only
Local clinical marker benchmark	C4 (inverse direction)	Local PBMC	−0.422	0.00079	0.731 (0.589–0.858)	—	Post hoc only
Local clinical marker benchmark	anti-dsDNA	Local PBMC	0.008	0.955	0.503 (0.338–0.675)	—	Uninformative for activity; post hoc only
IFN benchmark (score correlation)	IFN score vs compact score	GSE224705	0.102[1]	0.035	—	—	Weak; supports non-redundancy
IFN benchmark (disease activity)	IFN score	GSE224705	0.417	$<2\times10^{-16}$	0.733 (0.611–	0.783 (0.727–	Stronger than compact score; reported

Section	Readout	Cohort	SLEDAI ρ	SLEDAI P	High vs low/mod AUC (95% CI)	SLE/LN vs HC AUC	Note
					0.855)	0.839)	transparently
IFN benchmark (disease activity)	Compact score	GSE224705	0.189	8.69×10^{-5}	0.539 (0.417–0.660)	0.455 (0.370–0.540)	Weaker; captures complementary axis

[1]Patient-level sensitivity ρ = 0.019, P = 0.814. AUC 95% CIs by DeLong method. Abbreviations: AUC, area under ROC curve; CI, confidence interval; HC, healthy control; IFN, interferon; PBMC, peripheral blood mononuclear cell; SLE, systemic lupus erythematosus; SLEDAI, SLE Disease Activity Index.

Table S12. Post hoc single-gene and reduced-score benchmarking of the locked compact score panel.

All analyses are post hoc only. Gene-level z-scores and sign alignment followed the locked pre6-balanced protocol. Myeloid half-score: MMP9+MYL9+HAL (sign-aligned). Lymphoid half-score: CTLA4+CD40LG+VPREB3 (sign-aligned). Local PBMC: n = 60 SLE (high n = 42, low/moderate n = 18). Expanded whole-blood: n = 60 SLE (high n = 42, low/moderate n = 18). Results should not be used for retrospective panel revision; the panel was locked through public-stage adjudication before any local testing.

Readout	Cohort	SLEDAI ρ	SLEDAI P	High vs low/mod AUC	95% CI	Note	Analysis type
MMP9	Local PBMC	−0.056	0.671	0.513	NR	Weak in PBMC	Post hoc
MYL9	Local PBMC	0.277	0.037	0.678	NR	—	Post hoc
HAL	Local PBMC	−0.013	0.919	0.553	NR	—	Post hoc
CTLA4	Local PBMC	0.141	0.281	0.569	NR	—	Post hoc
CD40LG	Local PBMC	0.436	0.00050	0.753	NR	Strongest single-gene PBMC signal; not a new panel	Post hoc
VPREB3	Local PBMC	0.171	0.191	0.631	NR	—	Post hoc
Myeloid half-score	Local PBMC	0.062	0.638	0.592	NR	Weak in PBMC	Post hoc
Lymphoid half-score	Local PBMC	0.396	0.0018	0.764	NR	Not a replacement panel	Post hoc
Full 6-gene score (locked)	Local PBMC	0.281	0.029	0.719	0.571–0.844	Locked final score	Reference
MMP9	Expanded whole-blood RT-qPCR	0.617	<0.001	0.899	NR	Strong single-gene WB signal	Post hoc
MYL9	Expanded whole-blood RT-qPCR	0.387	0.002	0.656	NR	—	Post hoc
HAL	Expanded whole-blood RT-qPCR	0.376	0.003	0.709	NR	—	Post hoc

Readout	Cohort	SLEDAI ρ	SLEDAI P	High vs low/mod AUC	95% CI	Note	Analysis type
CTLA4	Expanded whole-blood RT-qPCR	−0.182	0.165	0.653	NR	—	Post hoc
CD40LG	Expanded whole-blood RT-qPCR	−0.513	<0.001	0.745	NR	—	Post hoc
VPREB3	Expanded whole-blood RT-qPCR	0.390	0.002	0.606	NR	—	Post hoc
Myeloid half-score	Expanded whole-blood RT-qPCR	0.711	<0.001	0.907	NR	Approaches full score	Post hoc
Lymphoid half-score	Expanded whole-blood RT-qPCR	0.100	0.446	0.628	NR	Weak in WB	Post hoc
Full 6-gene score (locked)	Expanded whole-blood RT-qPCR	0.819	1.25×10^{-15}	0.918	0.831–0.980	Locked final score; highest AUC and ρ in WB	Reference

NR = 95% CI not retained for this readout. All analyses are post hoc only. Spearman ρ among SLE samples. AUC for high vs low/moderate activity. Green rows = locked final score. Grey rows = reduced composites. Abbreviations: AUC, area under ROC curve; CI, confidence interval; PBMC, peripheral blood mononuclear cell; SLE, systemic lupus erythematosus; SLEDAI, SLE Disease Activity Index; WB, whole blood.
